# Robust Signal Processing in Living Cells

**DOI:** 10.1371/journal.pcbi.1002218

**Published:** 2011-11-17

**Authors:** Ralf Steuer, Steffen Waldherr, Victor Sourjik, Markus Kollmann

**Affiliations:** 1 Institute for Theoretical Biology, Humboldt University of Berlin, Berlin, Germany; 2 Manchester Interdisciplinary BioCentre, The University of Manchester, Manchester, United Kingdom; 3 Universität Stuttgart, Institute for Systems Theory and Automatic Control, Stuttgart, Germany; 4 Zentrum für Molekulare Biologie der Universität Heidelberg, DKFZ-ZMBH Alliance, Heidelberg, Germany; 5 Heinrich-Heine-Universität Düsseldorf, Department of Biology, Düsseldorf, Germany; University of Illinois at Urbana-Champaign, United States of America

## Abstract

Cellular signaling networks have evolved an astonishing ability to function reliably and with high fidelity in uncertain environments. A crucial prerequisite for the high precision exhibited by many signaling circuits is their ability to keep the concentrations of active signaling compounds within tightly defined bounds, despite strong stochastic fluctuations in copy numbers and other detrimental influences. Based on a simple mathematical formalism, we identify topological organizing principles that facilitate such robust control of intracellular concentrations in the face of multifarious perturbations. Our framework allows us to judge whether a multiple-input-multiple-output reaction network is robust against large perturbations of network parameters and enables the predictive design of perfectly robust synthetic network architectures. Utilizing the *Escherichia coli* chemotaxis pathway as a hallmark example, we provide experimental evidence that our framework indeed allows us to unravel the topological organization of robust signaling. We demonstrate that the specific organization of the pathway allows the system to maintain global concentration robustness of the diffusible response regulator CheY with respect to several dominant perturbations. Our framework provides a counterpoint to the hypothesis that cellular function relies on an extensive machinery to fine-tune or control intracellular parameters. Rather, we suggest that for a large class of perturbations, there exists an appropriate topology that renders the network output invariant to the respective perturbations.

## Author Summary

Cellular signaling networks have to function reliably and with high fidelity in an uncertain environment. In this paper, we investigate the topological principles to achieve such robust signal processing in living cells. Specifically, we identify the topological organizing principles that enable a signaling network to keep the stationary intracellular concentrations of certain molecules, such as active signaling compounds, within tightly defined bounds – despite conditions of uncertainty and in the face of multiple perturbations. We demonstrate that an appropriate topological organization renders the output of the pathway invariant against a large class of possible detrimental fluctuations, such as changes in energy states or total protein concentrations. Furthermore, we show that the topological requirements for robust signal processing can be formalized in terms of a linear vector space, denoted as invariant perturbation space, that predicts the robustness properties of the network. Constructing this invariant perturbation space for the *Escherichia coli* chemotaxis pathway reveals that the pathway is indeed invariant with respect to most dominant perturbations that would otherwise significantly hamper information transmission. Our framework provides a counterpoint to the hypothesis that cellular function relies on an extensive machinery to fine-tune or control intracellular parameters.

## Introduction

All living cells rely on the capacity to respond to intra- or extracellular signals and have evolved a dedicated biochemical machinery to continuously sense, transmit, and process a variety of internal and environmental cues. A key requisite for reliable signal processing is the capability of living cells to keep the stationary intracellular concentrations of certain molecules, such as active signaling compounds, within tightly defined bounds – despite conditions of uncertainty and in the face of multiple perturbations. While the apparent insensitivity of key intracellular concentrations, and hence of cellular function, to detrimental influences is widely recognized as a salient property of cellular signaling, knowledge of the precise mechanisms underlying these instances of pathway robustness is still fragmentary [1]–[6].

Here, we report a simple, yet highly efficient, novel formalism that pinpoints the necessary architecture for concentration robustness in living cells. We assert and substantiate by mathematical proof and experimental evidence that certain classes of network architectures render the functional output of the network, as represented by a set of steady state protein concentrations, invariant to a large class of perturbations. Our approach emphasizes robustness as a structural property of a network as a whole, rather than as a consequence of parameter-tuning or individual positive or negative interaction loops [3], [7], and offers a novel paradigm to understand the topological organization of cellular signaling networks. Differing from earlier approaches, our framework accounts for perturbations of large magnitude and is not restricted to a particular class of network kinetics, such as mass-action systems [5]. Applications include the robustness of input-output relationships with respect to variations in total component concentrations, reaction parameters, abundances of common resources like ATP, RNA polymerases, and ribosomes, as well as detrimental effects of pathway crosstalk, and variations in temperature. Our focus is on perturbations whose time scales are slow compared to the intrinsic dynamics of the pathway.

## Results/Discussion

### Local Concentration Robustness

To establish the mechanisms of robust signaling, we consider a multi input-multi output signaling network, whose temporal behavior is described by a set of ordinary differential equations for the state variables, 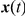, e.g., 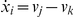, where the indices indicate different variables 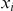 or reaction fluxes 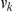. The equations can be organized into the more compact form,
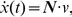
(1)where 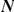 denotes the stoichiometric matrix. The reaction fluxes are specified by functions 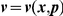 that depend on the variables 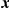 and a set of parameters 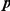. We require the existence of a – not necessarily unique – stationary state 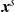 that obeys the steady state condition 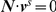 with 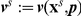. In the following, we assume that the functionality of the network is encoded in the steady state of a subset of output variables, defined as 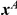, whose concentration values depend on a set of intra- or extracellular signals. The remaining intermediate variables are defined by 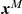. The system is said to exhibit *local concentration robustness* with respect to a particular parameter 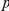 if a sufficiently small perturbation 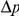 in this parameter does not affect the stationary concentrations of the output variables, 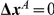. Mathematically, the perturbation is characterized by the vector of logarithmic partial derivatives 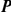 with elements 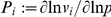, evaluated at the stationary state.

As the main result of the work, we now seek to identify stringent conditions on the network architecture – rather than on kinetic parameters – such that the robustness property holds for perturbations of large magnitude. To this end, we first recall the conditions for local concentration robustness. Utilizing results from linear control theory, local robustness can be ascribed to two scenarios: Either the perturbation has no effect on any stationary concentration within the network. In this case, the vector 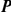 is an element of a vector space spanned by the columns of a matrix 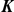 – with 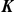 being a basis of the right nullspace of the scaled stoichiometric matrix, defined such that 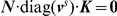. Or, more generally, the perturbation propagates through the network and affects the stationary concentration of some or all of the non-robust intermediate variables 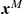, albeit without affecting the set of output variables 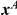. In this case, it can be shown that the perturbation vector 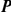 is an element of the joint vector space spanned by the columns of 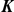 and the columns of a matrix 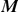. The latter matrix is given by the logarithmic partial derivatives of reaction rates with respect to the intermediate variables 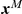, with elements 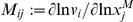. We note that the elements of 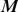 correspond to the *kinetic orders* or *scaled elasticities* of the reaction fluxes and attain integer values for the case of reaction networks that follow mass-action kinetics [8]. Taken together, a necessary and sufficient condition for local concentration robustness is therefore that the vector 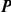 is an element of the vector space spanned by the columns of 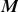 and 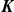, or equivalently, that the rank condition,
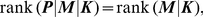
(2)is fulfilled. Here, the notation 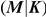 denotes a concatenation of the columns of both matrices. To ascertain local concentration robustness the rank condition is evaluated at the particular stationary state. See Materials and Methods and Text S1 for details and proof.

### From Local to Global Concentration Robustness

In general, local concentration robustness is not a sufficient condition to allow for robust signal processing in living cells. The fluctuations encountered by biological systems, such as variations in component concentrations arising from stochasticity in gene expression, are typically of large magnitude and cannot be described by local perturbations at a particular stationary state. Our aim is therefore to establish precise conditions for *global concentration robustness*. Specifically, a system is said to exhibit global concentration robustness with respect to a particular parameter 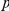 if the stationary concentrations of the set of output variables 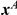 is invariant with respect to perturbations in 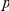. Thereby, 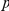 may take any value within a biophysically feasible perturbation set 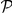 and is not restricted to small variations.

To obtain a viable criterion to judge global concentration robustness, we therefore extract from the local vector space, spanned by the columns of 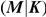, the largest subspace that does not depend on the choice of kinetic parameters, and hence, the specific stationary state. This subspace, denoted as the *invariant perturbation space* 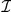, defines the largest vector space that guarantees local robustness at *any* stationary state of the system. Consequently, a perturbation of increasing magnitude that is confined to the invariant perturbation space may gradually affect the intermediate variables, but does not affect the designated output variables. The condition for global concentration robustness is then given by 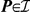, or, equivalently, as 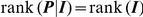, where 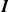 denotes a matrix whose columns span the vector space 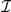.

We emphasize that the matrix 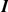 and its associated vector space are independent of kinetic parameters and therefore represent a genuine structural property of any signaling network. Proof and an algorithm is relegated to Materials and Methods and the SI, here we only outline its construction using a simple example.

### A Simple Example

**Figure 1:**
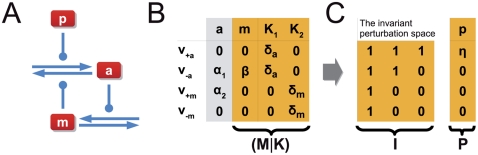
A simple example of global concentration robustness. (**A**) The output variable 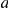 of the pathway is subject to a strong perturbation 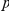 in its synthesis rate. Closed arrows denote regulatory interactions. (**B**) The concatenated matrix 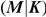 is constructed based on the network architecture. The first two columns correspond to the logarithmic partial derivatives of the rate equations with respect to both variables 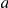 and 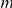. The latter two columns correspond to a representation of the scaled nullspace 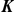. Greek letters denote unknown parameter-dependent values. (**C**) A largest parameter-independent representation 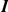, spanning the invariant perturbation space 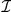, is obtained by elementary matrix operations. To test for output invariance, we ascertain that 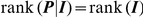, irrespective of kinetic parameters. The condition for global concentration robustness of 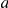 with respect to the perturbation p is thus fulfilled.

To illustrate the construction of the invariant perturbation space, we consider the simple pathway shown in Figure 1. Here, the output variable 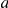 of the pathway is subject to strong fluctuations 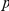 in its synthesis rate 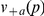. Rather than aiming to suppress the detrimental perturbations, the pathway employs an intermediate variable 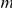 that compensates perturbations and ensures global concentration robustness of 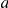. The pathway is described by two differential equations for the time-dependent behavior of the concentrations of 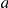 and 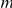, respectively,
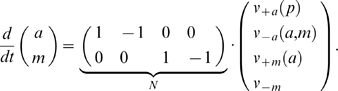
(3)For brevity, and as the only assumption on the rate equations and kinetic parameters, we require that the pathway gives rise to a unique stationary state for each value of 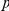. To obtain insight about the concentration robustness of the variable 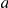 with respect to 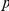, we construct the invariant perturbation space, derived from the concatenated matrix 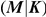. The matrix 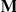 is given by the logarithmic partial derivatives of reaction rates with respect to the intermediate non-robust variable 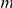. We obtain
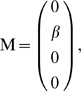
(4)where 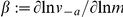 denotes the unknown state-dependent logarithmic partial derivative with respect to the variable 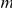. In general, the precise value of 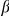 depends on the functional form of the rate equations, the value of the perturbation 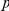, and the kinetic parameters.

The matrix 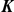 can be constructed algorithmically from the stoichiometric matrix. We obtain,
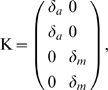
(5)where 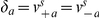 and 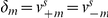 denote the stationary flux values.

To obtain a matrix representation 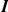 of the invariant perturbation space, we now need to identify the largest parameter-independent subspace spanned by the columns of 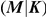. To this end, we note that the vector space spanned by the columns of a matrix remains invariant under elementary matrix operations (EMO), such as multiplication of a column by the same non-zero factor or the addition of an arbitrary multiple of one column to another. Applying a set of suitable EMOs, we obtain
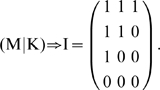
(6)We note that in this particular case, the invariant perturbation space is of the same dimension as the local vector space. In general, however, not all dimensions of the local space are retained, see Section III of Text S1 for an example.

To test for global concentration robustness of the variable 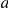 with respect to 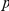, we now have to evaluate the rank condition 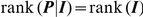. The perturbation is characterized by the vector
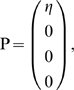
(7)where 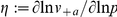 denotes the unknown state-dependent value of the logarithmic partial derivative. It can be straightforwardly ascertained that the rank condition for global concentration robustness is fulfilled, irrespective of the value of 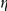. Hence, the variable 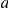 exhibits global concentration robustness with respect to perturbations in its synthesis rate.

We note that our simple example is a well-known instance of robust perfect adaptation [9], [10]. Biologically, the variable 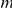 acts as an integrator, under the condition that the degradation rate of 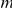 is independent of the concentration of 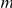 itself. Utilizing our approach, the invariant perturbation space can be constructed algorithmically for any given reaction network. The condition for global concentration robustness can then be ascertained by a simple numerical test and does not require extensive computations or additional expert knowledge.

### The Robustness of Two-Component Systems

To further illustrate the construction of the invariant perturbation space, we briefly consider the robustness of a canonical two-component system – one of the simplest and best-studied examples of robust signaling. Bacterial two-component systems typically consist of a membrane-bound sensor kinase that senses a specific stimulus and a cognate response regulator that modulates the signal response. Reliable functioning of two-component systems often requires that the output of the pathway, the concentration of phosphorylated response regulator as a function of an external stimulus, is not compromised by fluctuations in total protein concentrations of both components. The robustness of bacterial two-component systems with respect to such concentration fluctuations was investigated previously [11], [12]. In particular, Batchelor and Goulian [11] identified that the principal mechanism for concentration robustness is due to a bifunctional histidine kinase that phosphorylates and dephosphorylates its cognate response regulator.

**Figure 2:**
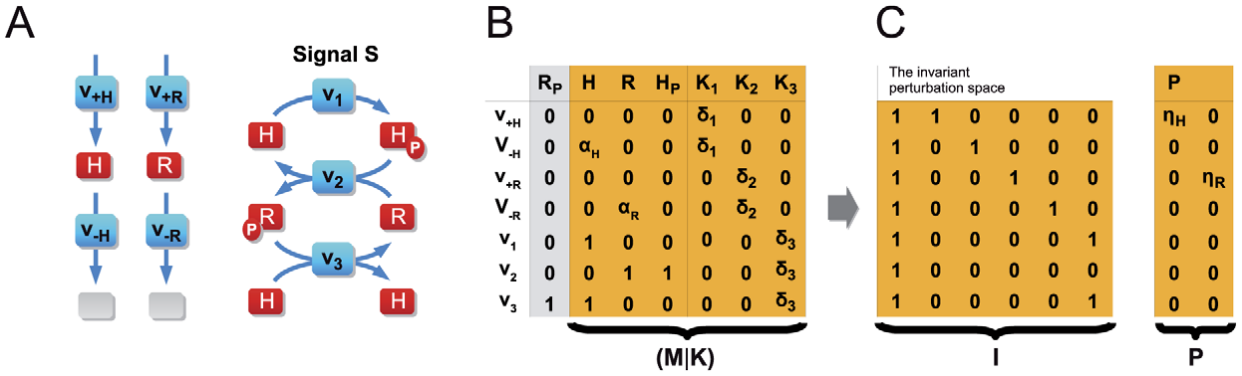
Robustness of two-component systems. (**A**) The model consists of 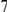 reaction rates and includes synthesis and degradation of the histidine kinase (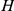) and the response regulator (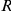). Robustness against fluctuations in expression is conveyed by the bifunctionality of the histidine kinase that catalyzes dephosphorylation of the response regulator (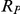). (**B**) The matrices 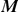 and 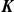 are constructed as described in the main text. Lowercase Greek letters denote real numbers, corresponding to unknown partial derivatives and unknown steady state reaction rates. (**C**) A matrix representation 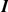 of the invariant perturbation space that is independent of kinetic parameters. The perturbations affect the synthesis rates of both proteins and the corresponding perturbation vectors have nonzero elements for the respective reaction rates. However, in both cases, the perturbation vector is an element of the invariant perturbation space, hence the condition for perfect concentration robustness of 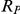 for these perturbations is fulfilled.

Figure 2 depicts a simplified model of the respective system. The histidine kinase (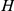) is phosphorylated by an external ligand. The phosphorylated kinase (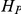) transfers the phospho-group to the unphosphorylated response regulator (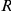). The pathway output is the concentration of the phosphorylated diffusible response regulator (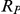). Importantly, dephosphorylation of the response regulator (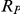) requires the participation of the bifunctional histidine kinase (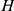). Utilizing our approach, we seek to confirm that, in this case, the stationary concentration of 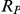 is invariant to variations in the expression levels of both proteins. For brevity, we again consider a highly simplified system and focus on the construction of the invariant perturbation space. In particular, the formation of protein complexes is neglected and all phosphorylation reactions are assumed to follow mass-action kinetics. A solution of the full system, including an explicit account of conserved moieties, is provided in Text S1 (Section VII).

To obtain the invariant perturbation space, we first derive the matrix 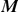 of logarithmic partial derivatives of reaction rates with respect to the non-robust variables 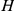, 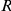, and 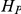. We assume that both proteins are synthesized and degraded with unknown rates 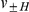 and 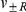 – using the simplifying assumption that degradation (or dilution) acts only on the unphosphorylated forms 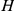 and 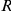. The unknown partial derivatives of the degradation reactions are denoted as 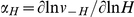 and 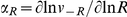, respectively. The remaining reactions are assumed to follow mass-action kinetics, resulting in partial logarithmic derivatives of unit value. Specifically, the phosphorylation rate 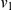 is dependent on the concentration of the unphosphorylated form 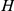, the phosphotransfer rate 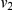 depends upon the concentration of 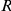 and 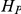, and the dephosphorylation rate 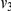 finally depends on the concentration of the phosphorylated response regulator 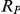, as well as the unphosphorylated form 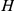 of the bifunctional kinase. The matrix 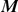 is given in Figure 2B.

As the next step, we need to identify the nullspace 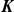 of the scaled stoichiometric matrix 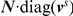. The nullspace of the unscaled stoichiometric matrix is readily available using standard tools of linear algebra. The representation of the unscaled nullspace is subsequently scaled with the unknown steady state reaction rates, such that 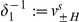, 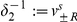, and 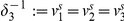. A representation of the scaled nullspace is provided in Figure 2B. Taken together, we again obtain the invariant perturbation space as the maximal subspace spanned by the columns of 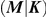 independent of kinetic parameters or steady state reaction rates. A matrix representation of the invariant perturbation space is given in Figure 2C.

We assume that the system is perturbed by unknown variations in the synthesis rates of both proteins, 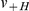 and 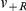, respectively. The corresponding partial derivatives with respect to unknown perturbations are denoted as 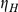 and 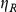 and shown in Figure 2C. To ascertain global concentration robustness of 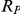, we confirm that the rank condition 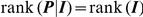 is indeed fulfilled. Hence, the output of the pathway, the steady state concentration of 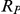, is invariant to perturbations in the synthesis rates of both components.

We note that, in general, our approach does presuppose that the system gives rise to a biologically feasible steady state solution for 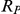. This requirement usually entails additional constraints on the possible reaction rates and kinetic parameters. For example, robustness of 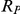 is only feasible under the condition that the total expression of the response regulator 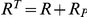 exceeds the steady state solution for 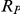. Below we present a generalization of the rank condition to account for additional constraints on molecule concentrations (see also Text S1, Section VIII).

### Conserved Moieties and Further Applications

Our approach is applicable to a variety of different scenarios, including several special cases which are discussed in the following. In particular, our approach relies on an interpretation of the elements of the matrix 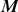 – the logarithmic partial derivatives of reaction rates with respect to the intermediate variables. For typical biochemical rate equations, these partial derivatives are nonlinear functions of kinetic parameters and therefore usually represent unknown and state-dependent quantities. However, as demonstrated above, our approach is still applicable in such a situation and does not require extensive knowledge of the functional form of the rate equations. In the most general case, each logarithmic partial derivative is represented by an unknown non-zero value within the matrix 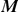. The resulting invariant perturbation space is required to be independent of these unknown derivatives. Hence, the invariant perturbation space is predominantly a structural property of the network and is identical for structurally equivalent networks. See Text S1 for details.

However, in some cases the elements of the matrix 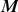 can be constraint further, owing either to particular functional forms of the rate equations or to simplifying assumptions that allow to approximate more complicated rate equations. An example of the former are generalized mass-action (GMA) kinetics of a reaction rate 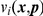,
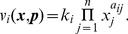
(8)For GMA kinetics, the partial logarithmic derivatives correspond to the exponents 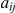 and are often considered to be constant quantities. Consequently, the partial logarithmic derivatives may be represented as constant entries within the matrix 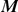. In this case, the invariant perturbation space is particularly straightforward to obtain.

As an example of simplifying assumptions, we note that complex rate equations are often approximated by more simple equations corresponding to specific kinetic regimes. In particular, a Michaelis-Menten equation can be approximated by a mass-action term or a constant for substrate concentrations far below or far above the Michaelis constant, respectively. In this case, the logarithmic partial derivative is approximately constant or zero, respectively. However, any result from applying the criterion for global concentration robustness is only valid as long as the assumptions underlying the approximation are fulfilled.

As yet, we have only considered reaction networks in the absence of mass-conservation relationships or conserved moieties. However, often the total concentration of some compounds can be considered as approximately constant over the relevant time-scales, giving rise to additional dependencies between variables. In this case, the system of differential equations for the *independent* state variables, 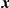 is augmented by a set of *dependent* state variables 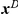, whose values are determined by a set of mass conservation equations. The full system of equations governing the time evolution of the system is
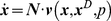
(9)

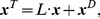
(10)with the vector 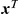 denoting the total concentration of each molecular component. The matrix 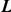 denotes a *link matrix* and usually consists of integer elements. To incorporate these dependencies within our approach, we must modify the definition of the matrix 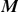 to account for the logarithmic partial derivatives with respect to the dependent variables. See Text S1 for details. Using the augmented matrix 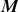, our approach proceeds as described above. As a corollary, we then obtain a simple criterion to judge global concentration robustness with respect to perturbations in conserved total concentrations [5], [6], see Text S1 (Section VII.B).

Our approach differs from a number of previous approaches to investigate robustness of biochemical reaction networks [1], [5], [6], [13]. The formalism is not restricted to systems described by mass-action kinetics, but is applicable a wide range of ODE-based descriptions of biochemical networks. Likewise, we do not focus on specific types of perturbations, such as variations in conserved moieties [5] or temperature [13]. Rather, our approach is applicable to any perturbation that can be described by a vector of partial derivatives of reaction rates – of which variations in conserved moieties, as well as of temperature are particular examples. We also mainly envision a scenario, where the perturbations are slow compared to the intrinsic fluctuation-compensation dynamics of the pathway. In particular, we consider the steady state of a selected subset of variables to represent the robust output of the system. Transient fluctuations in the vicinity of this state are not considered. However, the scenario described in this work indeed holds for many instances of cellular robustness. For example, in the case of gene expression noise, the observed fluctuations in expression levels are usually at least an order of magnitude slower than the phosphorylation dynamics in subsequent signaling pathways. Hence such fluctuations can be compensated by post-translational mechanisms – as described within this work. Similar arguments apply for several dominant fluctuations typically encountered by cellular signaling pathways, such as variations in temperature or abundance of common resources like ATP.

### The Robustness of the *Escherichia coli* Chemotaxis Pathway

**Figure 3:**
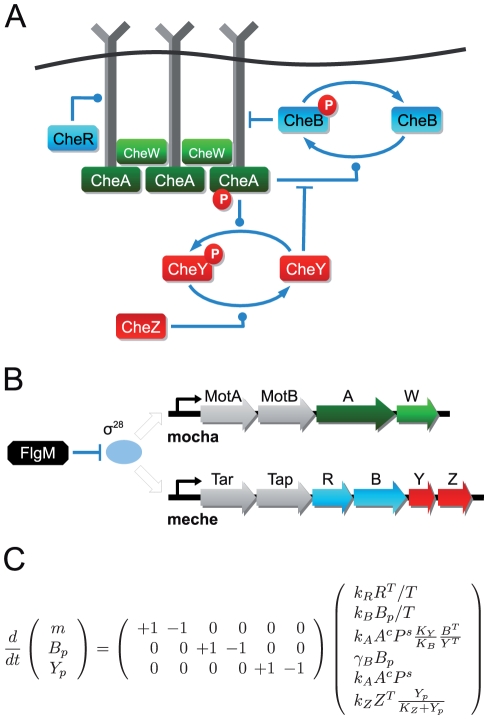
The *E. coli* chemotaxis pathway. (**A**) A pathway diagram and (**B**) the organization of its constitutive genes into two operons, denoted as 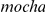 and 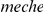. (**C**) To a good approximation, the pathway can be described by three variables: the average methylation state 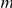, the concentration of phosphorylated methylesterases CheB (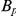) and the concentration of phosphorylated response regulator protein CheY (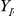). See Materials and Methods for definitions and equations.

To substantiate the explanatory power achieved by an interpretation of a complex cellular signaling network in terms of its associated invariant perturbation space, we now consider the robustness of the *E. coli* chemotaxis pathway. The topology of the pathway is depicted in Figure 3. The pathway responds to changes in concentrations of chemoeffectors such as certain amino acids or sugars by altering the phosphorylation state of the diffusible response regulator CheY. The concentration of free phosphorylated CheY (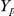) – the central output quantity of the pathway – then determines swimming behavior of the cell. Robust and precise regulation of 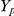 is a prerequisite for high chemotaxis efficiency and is maintained in the face of multifarious perturbations, most notably ATP availability, stochasticity in component abundance [14], and receptor cluster assembly [15], [16]. However, seemingly contradicting its functional objective, the pathway is rather sensitive to variations in the expression of some of its constituent proteins. For example, it was shown that a two-fold overexpression of CheZ or CheY levels already result in an 50% decrease of experimentally observed chemotactic performance, as determined by the size of swarm rings on soft agar plates [17].

**Figure 4:**
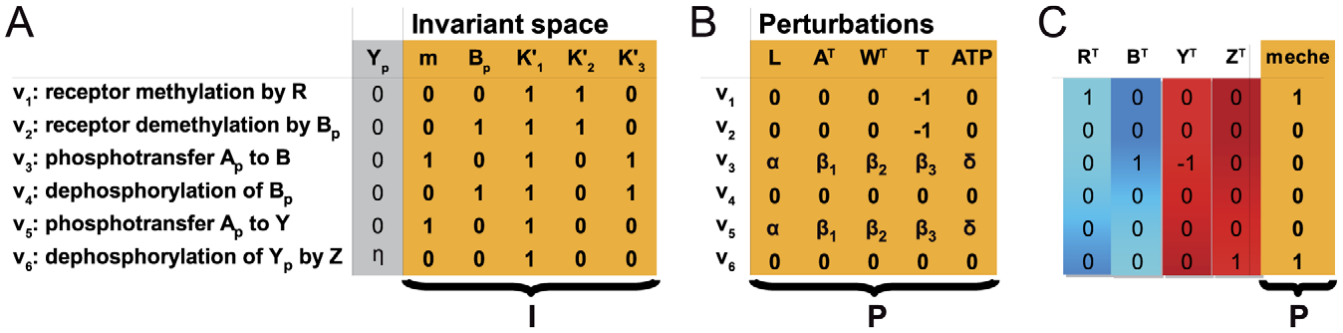
Robustness of the *E. coli* chemotaxis pathway. (**A**) A representation of the invariant perturbation space 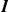, obtained from the concatenated matrix 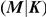. The column headers indicate the provenance of each column, as either a partial derivative with respect to the three variables 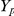, 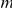, and 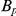, or the representation of the nullspace. (**B**) The perturbation vectors for variations in concentrations of chemoeffectors (L), total CheA (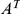), total CheW (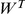), receptor assembly (T) and ATP availability (ATP). Lowercase Greek letters denote real numbers corresponding to contributions from the derivatives of (unspecified) nonlinear functions, namely 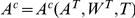, 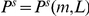, and 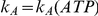. The rank condition, 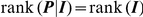, is fulfilled for each perturbation vector. Hence, the pathway output 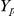 maintains global concentration robustness with respect to these perturbations. (**C**) Pertubations in the total concentrations of individual proteins CheR (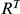), CheB (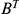), CheY (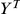), and CheZ (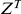) are *not* elements of the invariant space. However, the pathway exhibits robustness against concerted variations in the expression of the *meche* operon. In this case, the perturbation vector 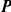 consists of additive contributions from each individual perturbation – corresponding to an effective reduction of dimensionality of the perturbations.

To reveal the mechanisms underlying the remarkable robustness that nonetheless allows reliable functioning of the pathway, we construct the invariant perturbation space 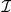 as described above. The concatenated matrix 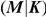 is obtained by considering the stoichiometric matrix and the kinetic dependencies shown in Figure 3. See SI (Section V) for details of the derivation. A parameter independent representation of the invariant perturbation space is shown in Figure 4A. To investigate the robustness of the pathway, we first consider changes in chemoeffector concentration (L), perturbations in the expression of CheA (A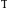) and CheW (W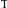), as well as variations in receptors (T) and ATP availability (ATP). The corresponding perturbation vectors are shown in Figure 4B. In each case, the corresponding perturbation vector is an element of the invariant perturbation space and the rank condition for global concentration robustness of 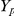 is fulfilled. Hence, the diffusible response regulator 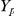 indeed exhibits global robustness of its stationary concentration with respect to these five highly detrimental influences.

**Figure 5:**
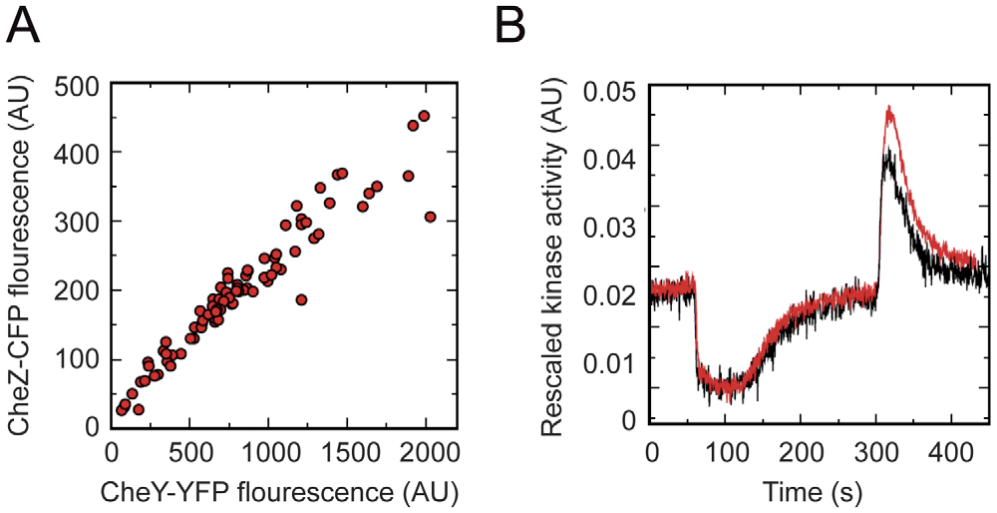
Concerted behavior of the expression level and robust response dynamics of the *E. coli* chemotaxis pathway as a consequence of the operon and regulon structure. (**A**) Single-cell concentrations of CheY-YFP and CheZ-CFP, bicistronically expressed from one plasmid pVS88 at 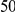 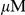 IPTG induction. (**B**) Response dynamics of the pathway activity measured by FRET after a step-like addition of attractant (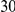 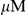 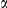-DL-methylaspartate) at time 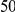 s, followed by attractant removal at time 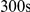, for native (black line) and seven fold upregulated (red line) transcriptional activity of the chemotaxis pathway genes (see SI, Section VI, for details).

Next, we consider changes in the expression of the individual proteins CheR (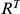), CheB (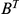), CheY (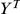), and CheZ (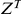). The corresponding perturbation vectors are given in Figure 4C. As can be ascertained by inspection of the rank condition, the respective perturbation vectors are *not* elements of the invariant space – in good agreement with the rather high sensitivity exhibited by the pathway in response to variations in the expression of these proteins [17]. Nonetheless, the observed total concentrations of CheR, CheB, CheY, and CheZ are not “fine-tuned” and are known to exhibit considerable variability under various conditions. To explain this alleged paradox, we have to take the sequential arrangement of genes into operons, as shown in Figure 3B, into account. A closer inspection of Figure 4 then reveals that perturbations that arise from *concerted* fluctuations in protein concentrations, induced by stochastic synthesis of *meche* operon transcripts, are within the invariant perturbation space. And, indeed, coupling of expression levels of chemotaxis proteins adjacent on an operon has been experimentally shown to positively correlate with chemotactic efficiency and to underlie active selection during chemotactic spreading on soft agar plates [18]. Generalizing from this example, we expect that gene organization into operons and expression from polycistronic mRNA is a generic, evolutionary driven, mechanism to alleviate detrimental effects of stochasticity in gene expression. In the context of our framework, coupling of expression on the transcriptional [14] and translational level [18], reduces the effective dimensionality of a perturbation, thereby enabling an invariant perturbation space of lower dimension to compensate and counteract the detrimental effects of fluctuations. In this sense, strong transcriptional and translational coupling is closely related to the robustness conveyed by bifunctional enzymes [5]. For the *E. coli* chemotaxis pathway strong coupling of genes expressed from one operon is evident in cells expressing yellow and cyan fluorescent protein fusions to CheY and CheZ, respectively, from one bicistronic plasmid construct, as shown in Figure 5A [14], [19]. The striking invariance of the pathway output upon a seven fold concerted increase in the transcriptional activity of the chemotaxis operons following the deletion of the anti sigma factor FlgM is shown in Figure 5B [14], [19].

As argued previously [20], the benefits of co-variation to reduce the effective dimensionality of perturbations are likely to confer a selective advantage strong enough to drive the assembly of genes into operons. Our results also highlight the functional importance of seemingly redundant or insignificant interaction characteristics, whose functional relevance is difficult to ascertain without an appropriate theoretical framework. A striking example is the catalyzed dephosphorylation of CheY by CheZ, as opposed to the uncatalysed dephosphorylation of CheB. While such a difference often seems extraneous to reliable signal transduction, such differences also shape the invariant perturbation space and are therefore crucial to achieve robust signal processing. A further example of a relevant interaction characteristic is the competitive binding of CheY and CheB to CheA, which results in a phosphotransfer rate to CheB that scales as 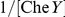. While not fine-tuned on the parameter level, this qualitative dependence is a prerequisite for robustness of the pathway output and in excellent agreement with experimental findings [21]. In this sense, our approach also offers a theoretical framework to investigate the functional relevance of given reaction characteristics – beyond their role in straightforward signal transmission.

### Conclusions

The interpretation of a complex cellular signaling network in terms of its associated invariant perturbation space has profound implications for our ability to understand and eventually rationally engineer robust biological circuits. There is increasing evidence that the utilization of post-transcriptional noise compensatory networks is a widespread mechanism in prokaryotic signaling. Experimentally ascertained examples include instances of two-component systems [1], [11], [12], the regulation of the glyoxylate bypass [22], and the sporulation network of *B. subtilis* [20]. In each case, an evolved network topology relegates potentially detrimental fluctuations in compound concentrations to its associated invariant perturbation space – rather than utilizing an expensive machinery to fine-tune native expression levels. We expect that similar mechanisms will provide an indispensable backbone for synthetic biology. Guided by the algorithmic construction of the invariant perturbation space, a key strategy for synthetic biology is to either maximize the invariant perturbation space by rationally rewiring the specificity of protein interactions [23], [24], or correlating perturbations among components, by placing genes on polycistronic mRNA or by building fusion constructs – in each case circumventing the need to fine-tune parameters that are experimentally hard to control. Our algorithm is applicable to large systems and requires only qualitative information on kinetic interactions. Our results allow us to clarify several long-standing issues relating to the emergence of cellular robustness. In particular, we hypothesize that the ubiquitous existence of puzzling, seemingly redundant, interaction loops that characterize our current understanding of cellular pathways is deeply rooted in as yet unrecognized mechanisms to counteract functional fragilities [10], [25]. In this sense, an interpretation of signalling architecture in terms of its invariant perturbation space offers a novel paradigm to understand cellular robustness, with the prospect to rationally engineer robust signaling circuits or target cellular defects.

## Materials and Methods

### Local Concentration Robustness

In the following, we outline the conditions for local concentration robustness, as stated in Eq. (2). We employ a logarithmic expansion of the stationary form of Eq. (1), 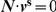, with 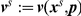, to linear order in a perturbation 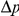 and the resulting changes in the state variables 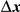,
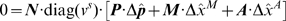
(11)with 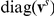 denoting a square matrix with entries 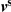 on the diagonal. The expansion coefficients are
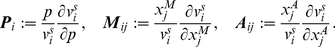
(12)The relative perturbation and its response are defined as 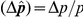, 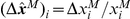, and 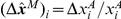.

In the absence of the condition for robustness of the pathway output, 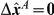, the expansion Eq. (11) has a unique solution for 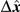 that quantifies the local linear response to a sufficiently small perturbation in parameters. The existence of the solution is guaranteed by the requirement that the Jacobian of the system is of full rank and hence invertible, implied by the dynamic stability of the considered steady state. Similar consideration are extensively utilized within, for example, Metabolic Control Analysis [8], [13], [26], [27].

However, the requirement of concentration robustness, 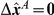, removes the degrees of freedom that correspond to (changes in) the output variables 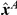. In this case, Eq. (11) translates into the condition
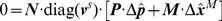
(13)In general, Eq. (13) is overdetermined, that is, no solution exists and the condition 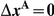 cannot be fulfilled. Eq. (13) has a unique solution 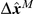 *if and only if* at least one of the following two conditions holds: Either the columns of the matrix 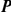 are elements of the right nullspace of the matrix 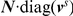, spanned by the columns of the matrix 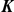. In this case, we obtain 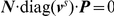 and, necessarily, 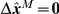. Or, the columns of the matrix 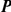 are linearly dependent on the columns of the matrix 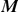. In mathematical terms, these two conditions can be summarized in the equation
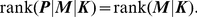
(14)Here, the columns of 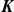 span the right nullspace of 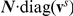, such that 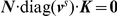. The notation 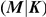 denotes a concatenation of the columns of the matrices 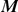 and 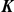, as described in the main text. See also SI (Sections II and IV) for a rigorous derivation.

### Towards Global Concentration Robustness

In the following, we outline the formal definitions and proof for global concentration robustness. For conciseness, we consider only generalized mass action (GMA) networks without conserved moieties. The general case, including a formal derivation of the conditions for global concentration robustness, is described in SI, Section IV. The biochemical network is defined as in Eq. (1). We consider a perturbation 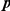 that takes values in a physically reasonable, connected set 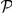. For a GMA network, the reaction rates are given by 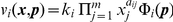 for reaction rates affected by the perturbation and 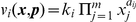 for reaction rates not affected by the perturbation. The concentration vector is split into 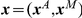 as described in the main text. The network is assumed to have a perturbation-dependent steady state 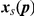 which is asymptotically stable for all 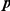 in a physically reasonable, connected perturbation set 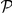.

The property of global concentration robustness is then formally defined as follows: For any values of the reaction rate parameters 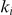 and any choice of the functions 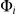, the steady state output concentration vector 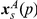 is constant over 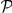.

The global invariant perturbation space as discussed in the main text for a GMA network is given by 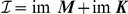, where 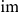 denotes the image or range of the matrix. Thereby, 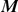 are the columns of the matrix with elements 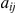, i.e. the logarithmic derivatives of the reaction rate vector with respect to 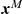, and 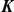 is a matrix whose columns span the space of the vectors which are in the kernel of 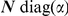 for all 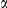 in the kernel of 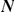.

To obtain a condition for global concentration robustness, we consider the vectors 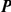 whose elements 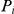 are zero whenever the reaction rate 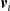 is not affected by the perturbation 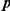. If all such vectors 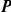 are element of the space 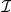, then the network has global concentration robustness. Conversely, if there exists such a 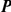 which is not in the space 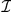, then there exists rate parameters 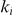 and functions 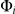 for which the steady state output concentration 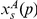 is not constant over 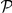, and thus the network does not have global concentration robustness. Computationally, the condition 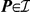 can be tested by the rank condition 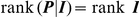, where 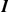 is any matrix whose columns span the space 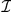.

### The *E. coli* Chemotaxis Pathway

The signal transduction of the *E. coli* chemotaxis pathway can be described to good accuracy by the interplay of the core components, the methyl accepting chemoreceptors (Tar, Tap, Tsr, Trg), the methyltransferase CheR, the methylesterase CheB, the response regulator CheY and its designated phosphatase CheZ (see Box 1). The total concentrations of these proteins are approximately 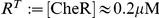, 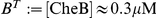, 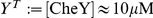, 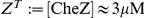, 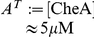, 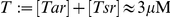, and 
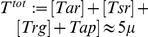
M. The concentration 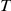 includes all receptors where CheR and phosphorylated CheB can bind to with high affinity, via a pentapeptide sequence at the carboxyl termini of the Tar and Tsr receptors. The set of mass action equations that determine the phosphorylation level of free diffusible response regulator proteins, 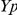, are listed below.

#### Methylation

The time evolution equation of the average receptor methylation level in the cell, 
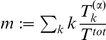
, with 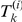 the concentration of receptors of type 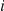 and 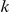 residues methylated, is given by
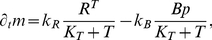
(15)with 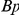 the concentration of phophorylated methylesterases, CheB, whose catalytic activity is 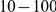-fold higher than in the unphosphorylated case. The dissociation constants of CheR and phosphorylated CheB to the pentapeptide sequence of Tar and Tsr are similar and are given a fixed value 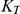 for both proteins. The functional form of the net methylation rate reflects experimental findings in the physiological relevant low-activity regime of receptor clusters [28]. We note that most mathematical models ignore CheB phosphorylation and assume that CheB acts predominantly on active receptors, a contribution which is ignored in our approach. As to leading order 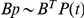, with 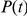 the probability to find receptors in the active state, both approaches show essentially the same adaptation dynamics. The reason why the net methylation rate does not follow the biochemically expected rate 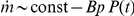 is still unknown [28].

#### Receptor activation

The signal amplification within a receptor cluster can be explained by assuming 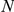 receptors to form independent allosteric units that change activity in unison [29]. Here, the probability to find an active receptor complex takes the form
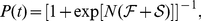
(16)with receptor energy 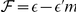, as a function of the average methylation level per receptor, 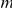, and the free energy contribution of attractant binding to receptors of type 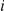, 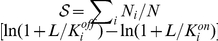, with 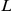 the ligand concentration. Any transient dynamics in receptor activation is absent for fixed 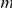 and 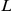 as the required conformational changes of these molecules equilibrate on the milliseconds time scale.

#### Binding of CheY to CheA

CheY binds with high affinity to the P2 domain of CheA with dissociation constants 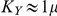
M, 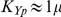
M and high on and off rates. This determines the free concentrations of CheA which is given by
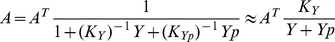
(17)Here, binding of CheB to CheA has been neglected as 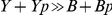.

#### Binding of CheB to CheA

CheB binds with high affinity to the P2 domain of CheA with dissociation constant 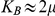
M and is assumed to have similar high on and off rates as CheY. This determines the free concentration of CheB given by
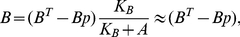
(18)where the approximation follows the same reasoning as above.

#### CheY phosphorylation

CheY receives phospho-groups at the P2 domain of CheA by phosphotransfer from the P1 domain of CheA. As P1 domain phosphorylation is the rate limiting step, only a small fraction of CheA is phosphorylated in the adapted state. We can therefore describe CheY phosphorylation dynamics to good approximation by
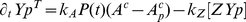
(19)

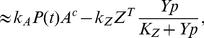
(20)where in the last line the 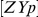 complexes have been resolved by introducing the Michaelis-Menten constant 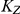. The concentration of total and free diffusible phosphorylated CheY is denoted by 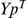 and 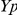, respectively. We emphasize that the autophosphorylation rate of CheA depends on the intracellular ATP concentration, 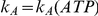, and only those P1 domains can be phosphorylated where CheA is part of functional allosteric receptor complexes. The concentration of these functional receptor-kinase complexes is denoted by 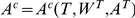 and depends on the concentrations of its constituents, CheA, CheW, Tar, Tap, Tsr and Trg, with variable receptor stoichiometry.

#### CheB phosphorylation

CheB gets phosphorylated at the P2 domain of CheA, receiving a phospho-group from the P1 domain of CheA. As for CheY, the P1 domain phosphorylation is believed to be the rate limiting step. Thus we have to good approximation
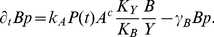
(21)Here, the term 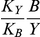 reflects the reduced phosphotransfer rate to CheB as a consequence of the 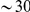-fold higher abundance of CheY, which occupies most of the P2 binding domains as 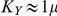
M.

### Stationary Solutions and the Dependency Matrices

In the following, we consider the stationary case of the chemotaxis equations. We thereby employ the approximations 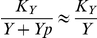 as 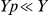, 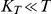, 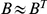 as 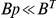, and 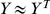. The simplified set of stationary equations read
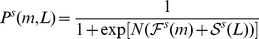
(22)

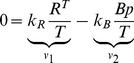
(23)

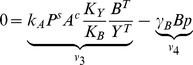
(24)

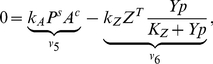
(25)where we have resolved the complexes 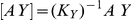 and 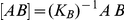 and introduced the stationary functions 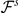 and 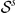 as defined above for time independent mean methylation level 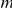 and fixed ligand concentration 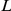. A derivation of the entries in Figure 4 is provided in Text S1.

## Supporting Information

Text S1 **Supplementary information.** A formal derivation of the conditions for global concentration robustness and additional examples.
(PDF)
