# Supplementary material for: Robust Signal Processing in Living Cells
Source: PLoS Comput Biol. 2011 Nov 17;7(11):e1002218. doi: 10.1371/journal.pcbi.1002218 (PMC3219616; doi:10.1371/journal.pcbi.1002218)
Supplement: Text S1 — Supplementary information. A formal derivation of the conditions for global concentration robustness and additional examples. (PDF) [file pcbi.1002218.s001.pdf]

Text S1 for 'Robust Signal Processing in Living Cells'

Contents

I. Introduction 2

II. Determination of the invariant perturbation space 3
- A. Biochemical networks without conserved moieties 3
- B. Biochemical networks with conserved moieties 6
- C. From local to global robustness: The invariant perturbation space 9
- D. Modelling biochemical reaction networks 11

III. Application of the formalism to a simple reaction network 13

IV. Formal derivation of the concentration robustness condition 17
- A. Notation 17
- B. Local concentration robustness 19
- C. Global concentration robustness 21

V. Robustness of the *Escherichia coli* chemotaxis pathway 26

VI. Materials and Methods 28
- A. Bacterial strains and plasmids 28
- B. Growth conditions 28
- C. FRET measurements 28
- D. Quantification of gene expression 28
- E. Note on Figure 4B 28

VII. Further Applications 30
- A. Two-component systems and implications for synthetic biology 30
- B. Conservation relationships and robustness of mass-action systems 36
- C. Complex perturbations and temperature compensation 37
- D. Robustness of bi- and multistable systems 39

VIII. Miscellanea 42
- A. Limitations of our approach 42
- B. Fine-tuned global robustness 42
- C. Numerical test of the rank condition 43
- D. Determination of the parameter-independent nullspace $\boldsymbol{K'}$ 44

References 47

I. INTRODUCTION

We aim at a mathematical formalism that allows to judge whether a multiple-input-multiple-output reaction network is robust against large perturbations of network parameters. In addition to identify design principles for robust signal processing, the formalism should indicate the necessary network modifications and extensions to arrive at a robust network output for specific perturbations, when starting from a non-robust network. In particular, our formalism builds upon the structural properties of a (bio)chemical network, as it is the network architecture and not 'fine-tuning' of parameters that allows for the compensation of large perturbations. Our results show that the robustness of a signaling network can be judged by inspection of a linear vector space: We demonstrate that for each biochemical network, there exists a linear vector space, such that any perturbation (expressed as a vector of partial logarithmic derivatives) that is confined to this vector space leaves the output of the network, as defined by a set of stationary concentrations of designated output variables, invariant. One of our main achievement is to identify which part of this vector space is independent of kinetic parameters (corresponding to global concentration robustness) and which part of this vector space is dependent on kinetic parameters (corresponding to local concentration robustness and requiring fine-tuning of parameters). The former is denoted as the *invariant perturbation space* of the network and can be algorithmically constructed for any signaling network.

Our framework provides a counterpoint to the hypothesis that cellular function relies on an extensive machinery to fine-tune or control intracellular parameters. Rather, our framework suggests that there exists an appropriate topology that renders the network output insusceptible to a given class of perturbations. Our framework draws upon and extends concepts of (metabolic) control theory. The reader familiar with nonlinear control theory will discover some parallels to the concept of *disturbance decoupling*. Within this Supplementary Information, we first outline our framework in more detail, point out pitfalls, and provide additional examples. A mathematical rigorous treatment is given in Section IV.

II. DETERMINATION OF THE INVARIANT PERTURBATION SPACE

A. Biochemical networks without conserved moieties

We first illustrate our concept using the special case of a biochemical network without conserved moieties. The biochemical network is assumed to consist of m independent dynamic state variables, $\boldsymbol{x} = (x_1, ..., x_m)$, whose temporal evolution is determined by a differential equation of the form,

$$\dot{\boldsymbol{x}} = \boldsymbol{N} \cdot \boldsymbol{v} \tag{1}$$

with $\boldsymbol{v} = (v_1, ..., v_k)$ a k-dimensional vector of reaction fluxes and $\boldsymbol{N}$ the stoichiometric matrix. We require that the rank of the stoichiometric matrix equals the number of state variables, $\text{rank}(\boldsymbol{N}) = m$, that is, $\boldsymbol{N}$ does not contain any linearly dependent rows. The functional forms of the reaction fluxes $\boldsymbol{v}(\boldsymbol{x}, \boldsymbol{p})$ describe the dependencies of reaction rates on compound concentrations and parameters. The latter may include a set of signals that represent the functional input of the system. At the most basic level, the rate equations are given by generalized mass-action (GMA) kinetics of the form

$$v_i(\boldsymbol{x}) = k_i \prod_{j=1}^{n} x_j^{\alpha_{ij}}, \tag{2}$$

with α_{ij} denoting the kinetic exponents that do not necessarily have to take integer values. With respect to the state variables, we further distinguish between a set of output state variables, defined as $\boldsymbol{x}^A$, and a set of intermediate state variables, $\boldsymbol{x}^{\boldsymbol{M}}$. As the prerequisite for robustness, we require that the output states are invariant under perturbations, that is, $\Delta \boldsymbol{x}^A = 0$, where $\Delta \boldsymbol{x}^A$ denotes the difference in the output variables after and before a perturbation Δp_j on network parameters.

In the following, we assume the existence of a (not necessarily unique) asymptotically stable stationary state $\boldsymbol{x}^{\boldsymbol{s}}$ with $\boldsymbol{N} \cdot \boldsymbol{v}(\boldsymbol{x}^{\boldsymbol{s}}) = \boldsymbol{0}$. We further assume that the functionality of the network is encoded in the stationary dependence of the designated output variables, $\boldsymbol{x}^{\boldsymbol{A}}$, on a set of input parameters.

We expand the stationary form of the Eq. (1), $\boldsymbol{N} \cdot \boldsymbol{v}^{\boldsymbol{s}} = \boldsymbol{0}$, with $\boldsymbol{v}^s := \boldsymbol{v}(\boldsymbol{x}^s)$, to linear order in both the perturbations, Δp_j, that change network fluxes $v_i^s(p_j) \rightarrow v_i^s(p_j + \Delta p_j)$ and the resulting changes in the state variables $\Delta \boldsymbol{x}$,

$$0 = \boldsymbol{N} \cdot \text{diag}(\boldsymbol{v}^s) \cdot \left(\boldsymbol{P} \cdot \Delta \hat{\boldsymbol{p}} + \boldsymbol{M} \cdot \Delta \hat{\boldsymbol{x}}^M + \boldsymbol{A} \cdot \Delta \hat{\boldsymbol{x}}^A \right) \tag{3}$$

with $\text{diag}(\boldsymbol{v}^s)$ denoting a square matrix with entries $\boldsymbol{v^s}$ on the diagonal and the expansion coefficients

$$\boldsymbol{P}_{ij} := \frac{p_j}{v_i^s}\frac{\partial v_i^s}{\partial p_j}, \quad \boldsymbol{M}_{ij} := \frac{x_j^M}{v_i^s}\frac{\partial v_i^s}{\partial x_j^M}, \quad \boldsymbol{A}_{ij} := \frac{x_j^A}{v_i^s}\frac{\partial v_i^s}{\partial x_j^A}. \tag{4}$$

The relative perturbations and its responses are defined as $(\Delta\hat{\boldsymbol{p}})_i = \Delta p_i/p_i$, $(\Delta\hat{\boldsymbol{x}}^M)_i = \Delta x_i^M/x_i^M$, and $(\Delta\hat{\boldsymbol{x}}^M)_i = \Delta x_i^A/x_i^A$. We note that if the reaction fluxes follow the functional form given in Eq. (2), the expansion coefficients are given by the constant kinetic coefficients α_{ij}.

In the absence of the condition $\Delta\hat{\boldsymbol{x}}^A = \boldsymbol{0}$, the expansion Eq. (3) always has a unique solution $\Delta\hat{\boldsymbol{x}}$ that quantifies the local linear response to an infinitesimal perturbation in parameters. The existence of the solution is guaranteed by the fact that the Jacobian of the system is of full rank and hence invertible – a condition that is extensively utilized within, for example, Metabolic Control Analysis [3, 7].

However, our requirement of robustness $\Delta\hat{\boldsymbol{x}}^A = \boldsymbol{0}$ removes those degrees of freedom that correspond to (changes in) the output variables $\hat{\boldsymbol{x}}^A$. As a consequence, only the set of intermediate variables $\hat{\boldsymbol{x}}^M$ is able to compensate the perturbations. In this case, Eq. (3) translates into the condition

$$\boldsymbol{N} \cdot \text{diag}(\boldsymbol{v}^s) \cdot \boldsymbol{P} \cdot \Delta\hat{\boldsymbol{p}} = -\boldsymbol{N} \cdot \text{diag}(\boldsymbol{v}^s) \cdot \boldsymbol{M} \cdot \Delta\hat{\boldsymbol{x}}^M \quad . \tag{5}$$

In general, Eq. (5) is overdetermined, that is, no solution exists, hence the condition $\Delta\boldsymbol{x}^{\boldsymbol{A}} = \boldsymbol{0}$ cannot be fulfilled. Eq. (5) has a unique solution $\Delta\hat{\boldsymbol{x}}^M$ *if and only if* at least one of the following two conditions holds: Either the columns of the matrix $\boldsymbol{P}$ are elements of the right nullspace of the matrix $\boldsymbol{N}\cdot\text{diag}(\boldsymbol{v}^s)$, with $\boldsymbol{N}\cdot\text{diag}(\boldsymbol{v}^s)\cdot\boldsymbol{P} = \boldsymbol{0}$. Then, necessarily, $\Delta\hat{\boldsymbol{x}}^M = \boldsymbol{0}$. Or, the columns of the matrix $\boldsymbol{P}$ are linearly dependent on the columns of the matrix $\boldsymbol{M}$. In mathematical terms, these two conditions can be summarized in the equation

$$\text{rank}(\boldsymbol{P}|\boldsymbol{M}|\boldsymbol{K}) = \text{rank}(\boldsymbol{M}|\boldsymbol{K}) \quad . \tag{6}$$

Here, the columns of $\boldsymbol{K}$ span the right nullspace of $\boldsymbol{N} \cdot \text{diag}(\boldsymbol{v}^s)$, such that $\boldsymbol{N} \cdot \text{diag}(\boldsymbol{v}^s) \cdot \boldsymbol{K} = \boldsymbol{0}$. The notation $(\boldsymbol{M}|\boldsymbol{K})$ denotes a concatenation of the columns of the matrices $\boldsymbol{M}$ and $\boldsymbol{K}$. Equation (6) expresses the condition that each column vector of the matrix $\boldsymbol{P}$ must be linearly dependent on the column vectors of $(\boldsymbol{M}|\boldsymbol{K})$.

We note that, considering again the full system in Eq. (3), the concatenated matrix $(\boldsymbol{A}|\boldsymbol{M}|\boldsymbol{K})$ is square and of full rank. This property again reflects the fact that there

exists a local linear response in the systems variables for any local perturbation in the rate equations – a consequence of the Jacobian being invertible. Conversely, the concatenation $(\boldsymbol{M}|\boldsymbol{K})$ is *not* of full rank, hence Eq. (6) cannot be fulfilled for arbitrary perturbations. However, if Eq. (6) is fulfilled, then the system indeed exhibits local concentration robustness, as defined by $\Delta\hat{\boldsymbol{x}}^A = \mathbf{0}$. Here, we emphasize that the linear system Eq. (3) has (locally) a unique solution. Hence, if a solution with $\Delta\hat{\boldsymbol{x}}^M \neq \mathbf{0}$ and $\Delta\hat{\boldsymbol{x}}^A = \mathbf{0}$ is identified as a possible solution of the system, it necessarily corresponds to the only solution of the linear perturbation problem.

In addition to the rank condition Equation (6) given above, an equivalent condition for local concentration robustness can be stated in terms of linear vector spaces. To this end, we denote by $colsp\,\boldsymbol{P}$ the column space of the matrix $\boldsymbol{P}$, that is, the vector space that is spanned by the columns of $\boldsymbol{P}$. Further, we define by $colsp(\boldsymbol{M}|\boldsymbol{K}) := colsp\,\boldsymbol{M} + colsp\,\boldsymbol{K}$ the joint linear vector space spanned by the columns of $\boldsymbol{M}$ and $\boldsymbol{K}$. An equivalent condition to Eq. (6) is then given by demanding all column vectors of $\boldsymbol{P}$ to be elements of this subspace $colsp\,\boldsymbol{P} \subseteq colsp(\boldsymbol{M}|\boldsymbol{K})$. A special case is given by $colsp\,\boldsymbol{P} \subset colsp\boldsymbol{K}$, that is a perturbation vector that is a subset of the nullspace necessarily implies perfect robustness of all state variables $\Delta\boldsymbol{x} = \mathbf{0}$ with respect to this perturbation. This fact is also known from Metabolic Control Analysis.

As yet, the conditions for local concentration robustness were derived with respect to infinitesimal perturbations at a particular state $\boldsymbol{x}^{\boldsymbol{s}}$. In general, the matrices $\boldsymbol{M}$ and $\boldsymbol{K}$ will depend on the particular state at which the expansion was performed – hence Eq. (6) does not represent a sufficient condition for global robustness. In the following, as one of the major achievement of our work, we will derive the architectural requirements on the network that ensure that Eq. (6) is fulfilled independent of the particular stationary state, hence the system allows for global concentration robustness in the face of perturbations of large magnitude. Prior to this step, we briefly extend our analysis to networks that incorporate mass-conservation relationships.

B. Biochemical networks with conserved moieties

Most models of biochemical networks exhibit conserved moieties that usually arise from an approximation of slowly changing components by constant quantities. In this case, the system of differential equations for the *independent* state variables, $\boldsymbol{x} = (x_1, ..., x_m)$ is augmented by a set of *dependent* state variables $\boldsymbol{x}^D$, whose values are determined by n mass conservation equations. The full system of equations governing the time evolution of the system is

$$\dot{\boldsymbol{x}} = \boldsymbol{N} \cdot \boldsymbol{v}\left(\boldsymbol{x}, \boldsymbol{x}^D\right) \tag{7}$$

$$\boldsymbol{x}^T = \boldsymbol{L} \cdot \boldsymbol{x} + \boldsymbol{x}^D \quad , \tag{8}$$

with the vector $\boldsymbol{x}^T = (x_1^T, x_2^T, ..., x_n^T)$ denoting the total concentration of each molecular component. The matrix $\boldsymbol{L}$ has dimension $n \times m$ and the vector $\boldsymbol{x}^D = \left(x_1^D, ..., x_n^D\right)$ denotes the state variables that are determined by the conservation equations. The differential form of the mass conservation equations, Eq. (8), is given by $\Delta\boldsymbol{x}^D = -\boldsymbol{L} \cdot \Delta\boldsymbol{x}$. With respect to the independent state variables $\boldsymbol{x}$ we again distinguish between a set of intermediate state variables $\boldsymbol{x}^M$ and the output state variables $\boldsymbol{x}^A$ each typically representing different protein modification states or protein complexes. As above, we assume the output states to be invariant under perturbations, hence $\Delta\boldsymbol{x}^A = 0$, and expand the stationary form of the Eq. (7) to linear order in both the perturbations Δp_j and the resulting changes on the intermediate states $\Delta\boldsymbol{x}_M$

$$0 = \boldsymbol{N} \cdot \mathrm{diag}(\boldsymbol{v}^s) \cdot \left(\boldsymbol{P} \cdot \Delta\hat{\boldsymbol{p}} + \boldsymbol{D} \cdot \Delta\hat{\boldsymbol{x}}^D + \boldsymbol{M}^D \cdot \Delta\hat{\boldsymbol{x}}^M\right) \quad , \tag{9}$$

with expansion coefficients

$$\boldsymbol{P}_{ij} = \frac{p_j}{v_i^s}\frac{\partial v_i^s}{\partial p_j}, \quad \boldsymbol{D}_{ij} = \frac{x_j^D}{v_i^s}\frac{\partial v_i^s}{\partial x_j^D}\Bigg|_{\boldsymbol{x}^M=const} , \quad \boldsymbol{M}_{ij}^D = \frac{x_j^M}{v_i^s}\frac{\partial v_i^s}{\partial x_j^M}\Bigg|_{\boldsymbol{x}^D=const} . \tag{10}$$

The relative perturbations and its responses are again defined as $(\Delta\hat{\boldsymbol{p}})_i = \Delta p_i/p_i$, $(\Delta\hat{\boldsymbol{x}}^D)_i = \Delta x_i^D/x_i^D$, and $(\Delta\hat{\boldsymbol{x}}^M)_i = \Delta x_i^M/x_i^M$. We emphasize that the changes in states $\Delta\hat{\boldsymbol{x}}^D$ are entirely determined by changes in the intermediate states, $\Delta\hat{\boldsymbol{x}}^M$, via the differential mass conservation equation. Consequently, the dependent state variables do not represent additional degrees of freedom within the system and are not able to compensate perturbations.

Rather, the associated matrix $\boldsymbol{D}$ must be considered as indirect perturbations on the network that are induced by changes $\Delta\boldsymbol{x}^M$.

In mathematical terms, we can use the differential mass conservation relationship to substitute changes in the dependent variables by changes in independent intermediate variables, $\Delta\hat{\boldsymbol{x}}^D = -\boldsymbol{L''}\Delta\hat{\boldsymbol{x}}^M$, where $\boldsymbol{L''}$ denotes a scaled link matrix such that

$$\boldsymbol{L''} = \mathrm{diag}(\boldsymbol{x}^D)^{-1} \cdot \boldsymbol{L'} \cdot \mathrm{diag}(\boldsymbol{x}^M) \tag{11}$$

and $\boldsymbol{L'}$ is obtained from $\boldsymbol{L}$ by removing those columns that correspond to output variables. The condition for invariance of the output Eq. (6) can then be written as

$$\mathrm{rank}(\boldsymbol{P}|\underbrace{\boldsymbol{M^D} - \boldsymbol{D}\cdot\boldsymbol{L''}}_{\boldsymbol{M}}|\boldsymbol{K}) = \mathrm{rank}(\underbrace{\boldsymbol{M^D} - \boldsymbol{D}\cdot\boldsymbol{L''}}_{\boldsymbol{M}}|\boldsymbol{K}). \tag{12}$$

Eq. (12) is the generalized rank condition for local invariance of the output variables with respect to infinitesimal perturbations. A general definition of the matrix $\boldsymbol{M}$ is thus given by

$$\boldsymbol{M} := \boldsymbol{M}^D - \boldsymbol{D}\cdot\boldsymbol{L''} \quad . \tag{13}$$

In absence of conservation equations, $\boldsymbol{L} = \boldsymbol{0}$, we obtain the identity $\boldsymbol{M} = \boldsymbol{M}^D$ and thus recover our previous result Eq. (6).

As observed in Eq. (13), mass conservation relationships induce additional elements (dependencies) in the matrix of partial derivatives – a consequence of the substitution of the dependent variables within the kinetic rate equations.

We illustrate this point with a simple example. Consider a canonical two-component signal transduction network in bacteria, e.g. the EnvZ/OmpR system, where the histidine kinase with total concentration Z^T gets autophosphorylated and transfers the phospho-group to the response regulator. The response regulator, with total concentration R^T, is in turn dephosphorylated proportional to the phosphatase activity of the histidine kinase, which is proportional to Z.

$$\begin{aligned} Z &\overset{v_1}{\to} Z_p \\ R + Z_p &\overset{v_2}{\to} Z + R_p \\ R_p + Z &\overset{v_3}{\to} R + Z \end{aligned} \tag{14}$$

The system of differential equations for the independent variables is given as

$$\frac{d}{dt}\begin{pmatrix} Z_P \\ R_p \end{pmatrix} = \underbrace{\begin{pmatrix} +1 & -1 & 0 \\ 0 & +1 & -1 \end{pmatrix}}_{\boldsymbol{N}} \cdot \underbrace{\begin{pmatrix} k_1\,Z \\ k_2\,Z_p\,R \\ k_3\,Z\,R_p \end{pmatrix}}_{\boldsymbol{v}} \quad . \tag{15}$$

The reaction network further satisfies the conservation equations

$$\begin{aligned} Z^T &= Z + Z_p \\ R^T &= R + R_p \end{aligned} \qquad \longrightarrow \qquad L = \begin{pmatrix} 1 & 0 \\ 0 & 1 \end{pmatrix} \quad , \tag{16}$$

using the assignments $\boldsymbol{x}^T = (Z^T, R^T)$, $\boldsymbol{x} = (Z_p, R_p)$ and $\boldsymbol{x}^D = (Z, R)$, and assuming R_p as the output variable of the pathway.

Utilizing the definitions given above, we then obtain

$$\boldsymbol{M}^{\boldsymbol{D}} = \left(\begin{array}{c|c} & Z_p \\ \hline v_1 & 0 \\ v_2 & 1 \\ v_3 & 0 \end{array}\right) \qquad \text{and} \qquad \boldsymbol{L}'' = \begin{pmatrix} Z_p/Z \\ 0 \end{pmatrix} \quad , \tag{17}$$

hence the matrix $\boldsymbol{M}$ is given as,

$$\boldsymbol{M} = \left(\begin{array}{c|c} & Z_p \\ \hline v_1 & 0 \\ v_2 & 1 \\ v_3 & 0 \end{array}\right) - \left(\begin{array}{c|cc} & Z & R \\ \hline v_1 & 1 & 0 \\ v_2 & 0 & 1 \\ v_3 & 1 & 0 \end{array}\right) \cdot \underbrace{\begin{pmatrix} -\alpha \\ 0 \end{pmatrix}}_{\boldsymbol{L}''} = \left(\begin{array}{c|c} & Z_p \\ \hline v_1 & \alpha \\ v_2 & 1 \\ v_3 & \alpha \end{array}\right) \quad , \tag{18}$$

using the definition $\alpha := -Z_p/Z = -Z_p/(Z^T - Z_p)$. As compared to the situation without mass conservation relationships the matrix $\boldsymbol{M}$ contains additional elements, corresponding to the implicit dependencies of the dependent variables. For example consider the element $(\boldsymbol{M})_1$, with

$$(\boldsymbol{M})_1 = \frac{\partial \ln \nu_1}{\partial \ln Z_p} = \frac{Z_p}{\nu_1}\frac{\partial\left(k_1(Z^T - Z_p)\right)}{\partial Z_p} = -\frac{Z_p}{Z} \quad . \tag{19}$$

In Section VII A of this Supplementary Information we will consider two-component systems and robustness against variation in total compound concentrations in more detail.

C. From local to global robustness: The invariant perturbation space

The basis of our approach is the transition from local concentration robustness to a criterion of global concentration robustness. In this respect, we require that the rank condition for local concentration robustness is fulfilled at any stationary state and irrespective of the kinetic parameters. Consequently, a perturbation that is large in magnitude may gradually alter the stationary state of the system by affecting the set of intermediate variables $\boldsymbol{x^M}$ – however, the stationary concentrations of the set of designated output variables $\boldsymbol{x^A}$ are not affected. We note that in the following, unless otherwise stated, we always assume that the system gives rise to a globally stable steady state for all parameters. As a consequence, we assume the Jacobian to be invertible at each point in state-space. For a more formal treatment see also Section IV.

The condition for local concentration robustness is given by Eq. (6),

$$\mathrm{rank}(\boldsymbol{P}|\boldsymbol{M}|\boldsymbol{K}) = \mathrm{rank}(\boldsymbol{M}|\boldsymbol{K}) \quad . \tag{20}$$

The task is to ascertain whether the equation is fulfilled independent of kinetic parameters. To this end, we recall that the rank of a matrix is unchanged under elementary matrix operations (EMO), which are: (i) the exchange of any two columns (rows), (ii) the multiplication of a column (row) by the same non-zero factor, (iii) the addition of an arbitrary multiple of one column (row) to another. Utilizing a series of EMOs, we aim to remove explicit parameter dependencies from Eq. (6), thus obtaining a global structural condition for concentration robustness.

To exemplify the application of EMO we continue with the example, a canonical two-component system, discussed in Eq. (14). In addition to the matrix $\boldsymbol{M}$, defined above, we construct the right nullspace $\boldsymbol{K}$ of the scaled stoichiometry, consisting of one column vector $\boldsymbol{K} = (\beta\ \beta\ \beta)^T$, with $\beta := (v_1)^{-1}$. We are interested in concentration robustness of the output variable R_p with respect to a perturbation p that affects both conserved total concentrations, $Z^T = Z^T(p)$ and $R^T = R^T(p)$. In this case, the perturbation vector reads

$$\boldsymbol{P} = \left(\begin{array}{c|c} & P \\ \hline v_1 & \gamma \\ v_2 & \delta \\ v_3 & \gamma \end{array}\right) \tag{21}$$

with $\gamma := \partial \ln v_1/\partial \ln p = \partial \ln v_3/\partial \ln p$, and $\delta := \partial \ln v_2/\partial \ln p$ (but see also subsequent sections for introductory examples). The rank condition is therefore given as

$$\operatorname{rank}\left(\begin{array}{c|c|cc} & P & Z_p & K \\ \hline v_1 & \gamma & \alpha & \beta \\ v_2 & \delta & 1 & \beta \\ v_3 & \gamma & \alpha & \beta \end{array}\right) = \operatorname{rank}\left(\begin{array}{c|cc} & Z_p & K \\ \hline v_1 & \alpha & \beta \\ v_2 & 1 & \beta \\ v_3 & \alpha & \beta \end{array}\right) \quad , \tag{22}$$

with $\boldsymbol{M}$ and α defined above. A parameter-independent representation is obtained by EMO

$$\left(\begin{array}{c|ccc} & P & Z_p & K \\ \hline v_1 & \gamma & \alpha & \beta \\ v_2 & \delta & 1 & \beta \\ v_3 & \gamma & \alpha & \beta \end{array}\right) \xrightarrow{EMO} \begin{pmatrix} 0 & 0 & 1 \\ 1 & 1 & 0 \\ 0 & 0 & 1 \end{pmatrix} \qquad \left(\begin{array}{c|cc} & Z_p & K \\ \hline v_1 & \alpha & \beta \\ v_2 & 1 & \beta \\ v_3 & \alpha & \beta \end{array}\right) \xrightarrow{EMO} \begin{pmatrix} 0 & 1 \\ 1 & 0 \\ 0 & 1 \end{pmatrix} \quad . \tag{23}$$

Obviously, the columns of $(\boldsymbol{M}|\boldsymbol{K})$ span a two dimensional plane that does not change its orientation in the three dimensional space under changes in $\boldsymbol{v}^s$, $\boldsymbol{x}^M$, and kinetic parameters that enter the equations via the expressions for α, β, γ, and δ. Thus any vector $\boldsymbol{P}$ that lies within this plane fulfills the rank condition. In the example considered here the vector $\boldsymbol{P} = (\gamma \quad \delta \quad \gamma)^T$ lies in the plane spanned by column vectors $(0 \quad 1 \quad 0)^T$ and $(1 \quad 0 \quad 1)^T$. Since the rank condition is fulfilled irrespective of the stationary state and kinetic parameters, the stationary network output, the variable R_p is globally robust with respect to $\boldsymbol{P}$.

However, in general not all dependencies on the stationary state of the matrix $(\boldsymbol{M}|\boldsymbol{K})$ can be removed by elementary matrix operations (EMO). We therefore define by $\mathcal{I}$ the space spanned by the largest possible set of parameter independent column vectors of $(\boldsymbol{M}|\boldsymbol{K})$,

$$\mathcal{I} = colsp(\boldsymbol{M}'|\boldsymbol{K}') \tag{24}$$

with $(\boldsymbol{M}'|\boldsymbol{K}')$ a reduced, maximally parameter free representation. We call $\mathcal{I}$ the invariant perturbation space. The invariant perturbation space contains exclusively structural information of a reaction network. Our approach therefore allows to separate the structural from fine tuned network properties. In terms of robustness – as defined in this work – this means that any perturbations $\boldsymbol{P}$ of the reaction fluxes that lie entirely in the invariant subspace result in an invariant system output. If we define by $(\boldsymbol{P}'|\boldsymbol{M}'|\boldsymbol{K}')$ the matrix resulting from

$(\boldsymbol{P}|\boldsymbol{M}|\boldsymbol{K})$ after appropriate elementary matrix operations, then

$$colsp\,\boldsymbol{P}' \subseteq \mathcal{I} \tag{25}$$

is a sufficient condition for structural robustness. For a rigorous definition see Section IV.

D. Modelling biochemical reaction networks

Our approach relies on an interpretation of the network structure in terms of the logarithmic partial derivatives of the kinetic rate equations. In particular, we utilize the fact that the logarithmic partial derivatives are – for certain kinetic functions – a genuine structural property of a reaction network.

At the most basic level kinetic rate equations are given by generalized mass-action functions of the form

$$v_i(\boldsymbol{x}) = k_i \prod_{j=1}^{n} x_j^{\alpha_{ij}}, \tag{26}$$

with the partial logarithmic derivatives given by

$$\frac{\partial \ln v_i}{\partial \ln x_j} = \frac{x_j}{v_i}\frac{\partial v_i}{\partial x_j} = \alpha_{ij} \qquad \text{and} \qquad \frac{\partial \ln v_i}{\partial \ln k_i} = \frac{k_i}{v_i}\frac{\partial v_i}{\partial k_i} = 1 \quad . \tag{27}$$

The partial logarithmic derivatives, corresponding the scaled elasticities of Metabolic Control Analysis (MCA), are restricted to constant (usually integer) values – corresponding to the *kinetic order* of each reaction with respect to its substrates.

We emphasize that many deterministic biochemical reaction networks can be described at the level of mass-action kinetics, such that all reaction equations comply with the functional form given above. In this case, and in the absence of mass conservation relationships, the columns of $\boldsymbol{M}$ are invariant under changes in rate constants and therefore already represent global structural properties of the reaction network.

However, sometimes the computational description of biochemical networks requires nonlinear equations – usually arising from approximations by rapid equilibrium or quasi steady state assumptions. In this case, the respective logarithmic partial derivatives are dependent on kinetic parameters and may take different values for different stationary states of the system. For example, for a generic Michaelis-Menten equation,

$$v(x) = \frac{V_m x}{K_M + x} \quad , \tag{28}$$

we obtain

$$\frac{\partial \ln v}{\partial \ln x} = \frac{x}{v}\frac{\partial v}{\partial x} = \frac{K_M}{K_M + x} \leq 1 \quad . \tag{29}$$

In this case the partial logarithmic partial derivative is a non-constant quantity that explicitly depends on kinetic parameters, as well as on the stationary state of the system. Nonetheless, our framework is also applicable in this case – as shown in the previous sections.

We note that the requirements for a given network to exhibit global concentration robustness depends to some extend on the interpretation of the logarithmic partial derivative. In particular, we can distinguish between two scenarios with respect to the interpretation of the logarithmic partial derivatives. Within the most strict assessment of global concentration robustness, we can assume that all partial derivatives are unknown and possibly variable quantities. This assumptions then also extend to generalized mass-action kinetics, such that respective logarithmic partial derivatives are not necessarily assumed to be constant quantities. In this case, the system exhibits concentration robustness also in the face of deviations from mass-action kinetics.

However, within a less stringent scenario – usually adopted within this work – we assume that the partial derivatives of mass-action rates are constant quantities that are part of the topology of the respective network. In this case, global concentration robustness may not be guaranteed for possible deviations from the assumed exponents.

We emphasize that the distinction described here does not affect or restrict the application of our framework to any actual topology – but rather it highlights that different assumptions on the nature of the rate equations may lead to different results with respect to global concentration robustness. See also Section IV for further analysis.

III. APPLICATION OF THE FORMALISM TO A SIMPLE REACTION NETWORK

To exemplify our formalism, we consider a simple example for output invariance, as shown in Fig. 1 of the main text. Here, an output variable a is subject to slow perturbations P in its synthesis rate. Rather than fine-tuning the value of p, we look for conditions, such that an intermediate variable m compensates perturbations and ensures perfect robustness with respect to the perturbation. The pathway is described by differential equations of the form

$$\frac{d}{dt}\begin{pmatrix} a \\ m \end{pmatrix} = \underbrace{\begin{pmatrix} +1 & -1 & 0 & 0 \\ 0 & 0 & +1 & -1 \end{pmatrix}}_{\boldsymbol{N}} \cdot \underbrace{\begin{pmatrix} v_{+a}(p) \\ v_{-a}(a,m) \\ v_{+m}(a) \\ v_{-m} \end{pmatrix}}_{\boldsymbol{v}} . \tag{30}$$

In the following, we require the system of differential equations to be well-defined, that is, all rate equations comply with basic assumptions about biochemical rate equations and the system gives rise to a positive steady state for any value of the perturbation P. Apart from these basic requirements, our method does not require to further specify the precise functional dependencies of the rate equations.

As depicted in Fig. 1 of the main text, the perturbation p acts on the rate v_{+a} with an (unspecified) nonlinear dependency $v_{+a} = v_{+a}(p)$. The elements of the perturbation vector $\boldsymbol{P}$ are defined as the logarithmic partial derivatives of the rate equations with respect to the perturbation. We use the abbreviation $\eta := \partial \ln v_{+a}/\partial \ln p$ and obtain

$$\boldsymbol{P} = \begin{pmatrix} \eta \\ 0 \\ 0 \\ 0 \end{pmatrix} . \tag{31}$$

We emphasize that our analysis does not require knowledge of the precise value of η, which usually depends on the specific functional form of the rate equations, the kinetic parameters, and the strength of the perturbation.

To obtain a condition for perfect robustness of the variable a with respect to variations in p, we follow the steps described in the main text. First, we determine the basis vectors of

the nullspace of $\boldsymbol{N} \cdot diag(\boldsymbol{v}^s)$. In practice any linear algebra software can be employed, such as the built-in function `null(N)` in matlab (The MathWorks). These basis vectors form the columns of the matrix $\boldsymbol{K}$. For the simple example, Eq. (30), the space spanned by the columns of $\boldsymbol{K}$ is given by

$$\boldsymbol{K} = diag(\boldsymbol{v}^s)\,\boldsymbol{K}^N = \begin{pmatrix} \delta_1 & 0 \\ \delta_1 & 0 \\ 0 & \delta_2 \\ 0 & \delta_2 \end{pmatrix} . \tag{32}$$

with $\delta_1 = (v_1^s)^{-1} = (v_2^s)^{-1}$ and $\delta_2 = (v_3^s)^{-1} = (v_4^s)^{-1}$.

Next, we consider the matrix $\boldsymbol{M}$, with elements corresponding to the logarithmic partial derivatives of the rate equations with respect to the variable m. With the abbreviation $\beta := \partial \ln v_{-a}/\partial \ln m$, evaluated at the stationary state, we obtain

$$\boldsymbol{M} = \begin{pmatrix} 0 \\ \beta \\ 0 \\ 0 \end{pmatrix} . \tag{33}$$

We note that here the reactions $v_{\pm m}$ do only depend on the output variable a and *not* on the variable m – a well-known prerequisite for perfect adaptation. Finally, we can assemble the invariant subspace, $\mathcal{I} = col(\boldsymbol{M}'|\boldsymbol{K}')$, using elementary matrix operations (EMO)

$$(\boldsymbol{M}|\boldsymbol{K}) = \left(\begin{array}{c|cc} 0 & \delta_1 & 0 \\ \beta & \delta_1 & 0 \\ 0 & 0 & \delta_2 \\ 0 & 0 & \delta_2 \end{array}\right) \overset{EMO}{\longrightarrow} (\boldsymbol{M'}|\boldsymbol{K'}) = \left(\begin{array}{c|cc} 0 & 1 & 0 \\ 1 & 1 & 0 \\ 0 & 0 & 1 \\ 0 & 0 & 1 \end{array}\right) . \tag{34}$$

A parameter-independent representation $\boldsymbol{I}$ of the invariant perturbation space $\mathcal{I}$ is given by

$$\boldsymbol{I} = \begin{pmatrix} 1 & 1 & 1 \\ 1 & 1 & 0 \\ 1 & 0 & 0 \\ 1 & 0 & 0 \end{pmatrix} . \tag{35}$$

In general the matrix representation $\boldsymbol{I}$ of the invariant perturbation space is not unique.

Any perturbation vector, $\boldsymbol{P}$, that is element of the invariant perturbation space does not

affect the stationary output of the system. Output invariance is tested by the rank condition, $rank(\boldsymbol{P}|\boldsymbol{I}) = rank(\boldsymbol{I})$, that reads – after performing elementary matrix operations to remove the unknown functional dependencies – as

$$\text{rank}\left(\begin{array}{c|ccc} 1 & 1 & 1 & 1 \\ 0 & 1 & 1 & 0 \\ 0 & 1 & 0 & 0 \\ 0 & 1 & 0 & 0 \end{array}\right) = \text{rank}\begin{pmatrix} 1 & 1 & 1 \\ 1 & 1 & 0 \\ 1 & 0 & 0 \\ 1 & 0 & 0 \end{pmatrix} , \tag{36}$$

The rank condition shows that the condition for global robustness of the output variable, a, is fulfilled. The example implements an integral feedback mechanism to realize a perfectly adapting reaction system. Here, the intermediate variable m acts as the 'integrator', with the crucial requirement that the rate of change of m is independent of the variable m itself. In our example, this implies that the rates $v_{\pm m}$ do *not* depend on the variable m (or, equivalently, that the dependence is with the same order) – otherwise perfect robustness cannot be achieved.

Indeed, we can give a counterexample to robust behavior if we assume that the degradation rate $v_{-m} = v_{-m}(m)$ is a function of m. In this case, Eq. (33) is modified and now reads

$$\boldsymbol{M} = \begin{pmatrix} 0 \\ \beta \\ 0 \\ \gamma \end{pmatrix} , \tag{37}$$

with $\gamma := \partial \ln v_{-m}/\partial \ln m$ denoting the unknown nonzero derivative. We obtain

$$(\boldsymbol{M}|\boldsymbol{K}) = \left(\begin{array}{c|cc} 0 & \delta_1 & 0 \\ \beta & \delta_1 & 0 \\ 0 & 0 & \delta_2 \\ \gamma & 0 & \delta_2 \end{array}\right) . \tag{38}$$

Since β and γ are both unknown and variable quantities, a parameter-independent representation $\boldsymbol{I}$ of the invariant perturbation space is restricted to two dimensions,

$$\boldsymbol{I} = \begin{pmatrix} 1 & 1 \\ 1 & 1 \\ 1 & 0 \\ 1 & 0 \end{pmatrix} . \tag{39}$$

In this case, the rank condition does not hold,

$$\text{rank}\begin{pmatrix} 1 & 1 & 1 \\ 0 & 1 & 1 \\ 0 & 1 & 0 \\ 0 & 1 & 0 \end{pmatrix} \neq \text{rank}\begin{pmatrix} 1 & 1 \\ 1 & 1 \\ 1 & 0 \\ 1 & 0 \end{pmatrix} \quad , \tag{40}$$

therefore the variable a does not exhibit global robustness.

IV. FORMAL DERIVATION OF THE CONCENTRATION ROBUSTNESS CONDITION

A. Notation

In this section, we provide a rigorous mathematical derivation of the conditions for local and global concentration robustness as obtained in Section II of the SI. Thereby, we consider biochemical network models with conserved moieties, and with reaction rates that may be a mixture of generalized mass action (GMA) with fixed exponents and arbitrary elements. First, in Section IV B, we derive the previously described rank condition as sufficient and necessary condition for local concentration robustness. Then, in Section IV C, to deal with the problem of global concentration robustness, we define the invariant perturbation space such that it is independent of the network's parameters and the non-GMA part of the reaction kinetics. Based on this definition, we derive a sufficient and (in a structural sense) necessary condition for global concentration robustness.

For ease of notation, we will restrict this section to a scalar perturbation. However, this is not a restriction of generality: if multiple perturbations are present, the condition can be evaluated for each perturbation individually, and the network is robust against combined perturbations if and only if it is robust against each perturbation individually.

The image of a matrix A is denoted by $\operatorname{im} A$, its kernel by $\ker A$. A diagonal matrix with diagonal entries taken from the components of a vector a is denoted by $\operatorname{dg} a$. The sum of two subspaces W_1 and W_2 of $\mathbb{R}^n$ is defined by

$$W_1 + W_2 = \{w_1 + w_2 \mid w_1 \in W_1, w_2 \in W_2\}. \tag{41}$$

A biochemical network model with conserved moieties is given by the differential algebraic equations

$$\begin{aligned} \dot{x} &= Nv(x, x^D, p) \\ x^T(p) &= Lx + x^D. \end{aligned} \tag{42}$$

Thereby, $x \in \mathbb{R}^m$ is the vector of independent concentrations, $N \in \mathbb{R}^{m\times k}$ the stoichiometric matrix, and v the reaction rate vector. For the conserved moieties, we have $x^T \in \mathbb{R}^n$ as the vector of total concentrations, while x^D is the vector of dependent state variables, related to the independent state variables x via the link matrix $L \in \mathbb{R}^{n\times m}$ [3]. We consider the effect of a perturbation to the network via the variable $p \in \mathbb{R}$, which is element of a connected and

open set $\mathcal{P} \subset \mathbb{R}$. Note that we have explicitly accounted for the possibility that the vector of total concentrations x^T may depend on the perturbation variable p. The model (42) can be transformed to an equivalent differential equation by substituting the dependent variables, yielding

$$\dot{x} = Nv(x, x^T(p) - Lx, p). \tag{43}$$

The species vector x is split into output variables and intermediate variables by writing

$$x = \begin{pmatrix} x^A \\ x^M \end{pmatrix}, \tag{44}$$

where $x^A \in \mathbb{R}^{m^A}$ are the output variables and $x^M \in \mathbb{R}^{m^M}$ are the intermediate variables. According to this splitting, we introduce the matrices $J_A, J_M \in \mathbb{R}^{m\times m}$ given by

$$J_A = \begin{pmatrix} I_{m^A} & 0 \\ 0 & 0 \end{pmatrix} \qquad J_M = \begin{pmatrix} 0 & 0 \\ 0 & I_{m^M} \end{pmatrix}, \tag{45}$$

where I_m is the identity matrix of dimension m, yielding

$$\begin{pmatrix} 0 \\ x^M \end{pmatrix} = J_M x \quad \text{and} \quad \begin{pmatrix} x^A \\ 0 \end{pmatrix} = J_A x. \tag{46}$$

We assume throughout that there exists a perturbation-dependent positive steady state in the independent variables x, given by a function $x_s : \mathbb{R} \to \mathbb{R}^m_+$, such that

$$Nv(x_s(p), x^T(p) - Lx_s(p), p) = 0. \tag{47}$$

The steady state map for the dependent state variables is defined as

$$x^D_s(p) := x^T(p) - Lx_s(p). \tag{48}$$

For ease of notation, we define

$$\bar{v}(p) := v(x_s(p), x^D_s(p), p) \tag{49}$$

as a shortcut for the steady state reaction rates.

Throughout this section, we assume that the steady state $x_s(p)$ is asymptotically stable for all $p \in \mathcal{P}$. Note that this implies that the Jacobian of the right hand side of (43) in steady state,

$$N\frac{\partial v}{\partial x}(x_s(p), x^D_s(p), p) - N\frac{\partial v}{\partial x^D}(x_s(p), x^D_s(p), p)L \tag{50}$$

is invertible for each $p \in \mathcal{P}$.

B. Local concentration robustness

Local concentration robustness at a perturbation value p is defined as the property that the derivative of the steady state output variables x_s^A with respect to the perturbation is equal to zero at p. The formal definition is given as follows.

Definition 1. *The network* (42) *is said to have local concentration robustness at* $p \in \mathcal{P}$*, if*

$$J_A x_s'(p) = 0. \tag{51}$$

In order to derive a necessary and sufficient condition for local concentration robustness, we introduce the following matrices:

$$\begin{aligned}
P(p) &= (\operatorname{dg} \bar{v}(p))^{-1} \Big(\frac{\partial v}{\partial p}(x_s(p), x_s^D(p), p) + \frac{\partial v}{\partial x^D}(x_s(p), x_s^D(p), p) \frac{\partial x^T}{\partial p}(p) \Big) p \\
Q(p) &= (\operatorname{dg} \bar{v}(p))^{-1} \frac{\partial v}{\partial x}(x_s(p), p) \operatorname{dg} x_s(p) \\
D(p) &= (\operatorname{dg} \bar{v}(p))^{-1} \frac{\partial v}{\partial x^D}(x_s(p), p) \operatorname{dg} x_s^D(p) (\operatorname{dg} x_s^D(p))^{-1} L \operatorname{dg} x_s(p) \\
M(p) &= (Q(p) - D(p)) J_M.
\end{aligned} \tag{52}$$

With these definitions, we next introduce the invariant perturbation space, a central network characteristic for concentration robustness. In words, the invariant perturbation space is the vector space of all infinitesimal directions in which perturbations can act on the steady state reaction rates without affecting the concentration values in steady state. The formal definition makes use of the matrices defined in (52) and is given as follows.

Definition 2. *The space*

$$\mathcal{I}(p) = \operatorname{im} M(p) + \ker(N \operatorname{dg} \bar{v}(p)). \tag{53}$$

is called the local invariant perturbation space of the network (42) *at* p.

We then have the following result as a condition for local concentration robustness of network (42).

Theorem 1. *The network* (42) *has local concentration robustness at* $p \in \mathcal{P}$*, if and only if*

$$P(p) \in \mathcal{I}(p). \tag{54}$$

Proof. First, from differentiating the steady state equation (47) with respect to p we obtain

$$N\frac{\partial v}{\partial x}(x_s(p), x_s^D(p), p)x_s'(p)+N\frac{\partial v}{\partial x^D}(x_s(p), x_s^D(p), p)\big(\frac{\partial x^T}{\partial p}(p)-Lx_s'(p)\big)+N\frac{\partial v}{\partial p}(x_s(p), x_s^D(p), p) = 0. \tag{55}$$

We denote

$$H(p) = (\operatorname{dg} x_s(p))^{-1}x_s'(p)p$$

and observe that $J_A x_s'(p) = 0$ if and only if $J_A H(p) = 0$. With the definitions from (52), (55) is equivalent to

$$N \operatorname{dg} \bar{v}(p)\big((Q(p) - D(p))H(p) + P(p)\big) = 0. \tag{56}$$

Making use of the fact that $J_A + J_M = I$, we rewrite (56) as

$$N \operatorname{dg} \bar{v}(p)\big((Q(p) - D(p))J_A H(p) + M(p)H(p) + P(p)\big) = 0. \tag{57}$$

Necessity. Under the condition that $J_A H(p) = 0$, we find that

$$N \operatorname{dg} \bar{v}(p)M(p)H(p) = -N \operatorname{dg} \bar{v}(p)P(p). \tag{58}$$

All $P(p)$ which solve this equation are given by

$$P(p) = -M(p)H(p) + a_1, \tag{59}$$

with $a_1 \in \ker(N \operatorname{dg} \bar{v}(p))$, which implies (54).

Sufficiency. The condition (54) implies that we can write $P(p)$ as

$$P(p) = a_1 + \big(Q(p) - D(p)\big)a_2, \tag{60}$$

with $a_1 \in \ker(N \operatorname{dg} \bar{v}(p))$ and $a_2 \in \operatorname{im} J_M$. Thus, one particular $H(p)$ which solves (56) is such that

$$\begin{aligned} J_A H(p) &= 0 \\ J_M H(p) &= -a_2. \end{aligned} \tag{61}$$

Since the Jacobian (50) is invertible, (56) has a unique solution $H(p)$ for each $P(p)$, which is given by (61). This proves local concentration robustness. □

C. Global concentration robustness

In the next step, we turn to the property of global concentration robustness. Essentially, a network is said to have global concentration robustness if the output variables x^A in steady state are constant over the perturbation set $\mathcal{P}$. In addition, we are interested in a characterization of global concentration robustness which is given by the network structure alone, and does not depend on exact parameter values and specific functions, for example Michaelis-Menten or Hill kinetics, for generic reaction rates.

To this end, we separate reaction rates into a mass action and a generic part. Thereby, the characterization of global concentration robustness should not depend on the rate constants of the mass action part nor the exact choice of mathematical function of the generic part. However, the characterization may depend on the interaction structure of the network, i.e. which concentrations affect which reaction rate, and the stoichiometric coefficients of the mass action part entering the reaction rate expression as exponents, which are both attributed to the structure of the reaction network. Thus, in the following, the reaction rate vector v is assumed to be composed by elements of the form

$$v_i(x, x^D, p) = \Phi_i(x, x^D, p) \prod_{j=1}^{m} x_j^{a_{ij}} \prod_{j=1}^{n} (x_j^D)^{a_{ij}^D}, \tag{62}$$

with $i = 1, \ldots, k$, where Φ_i represents the generic part of the reaction rate and also includes the rate constant for the mass action part, and the rest represent the concentration dependent terms in the mass action part, with stoichiometric coefficients a_{ij} and a_{ij}^D.

Before considering global concentration robustness, we first introduce the weaker notion of concentration invariance, i.e. the property that the output concentrations are constant over $\mathcal{P}$, but not necessarily independent of network parameter values and choice of generic reaction rate expressions.

Definition 3. *The network* (42) *is said to have global concentration invariance, if* $x_s^A(p) = \bar{x}_s^A$*, a constant value, for all* $p \in \mathcal{P}$.

Corollary 1. *The network* (42) *has global concentration invariance, if and only if*

$$P(p) \in \mathcal{I}(p) \tag{63}$$

for all $p \in \mathcal{P}$.

Proof. Since $\mathcal{P}$ is connected and x_s is assumed to be a continuously differentiable function over $\mathcal{P}$, the condition that $x_s^A(p)$ is constant over $\mathcal{P}$ is equivalent to

$$J_A x_s'(p) = 0 \tag{64}$$

for all $p \in \mathcal{P}$. Then, the result is a direct consequence of Theorem 1: if (63) is satisfied, Theorem 1 assures that (64) holds for any $p \in \mathcal{P}$, thus we have global concentration invariance. Conversely, if $P(\tilde{p}) \notin \mathcal{I}(\tilde{p})$ for some $\tilde{p} \in \mathcal{P}$, then by Theorem 1 it follows that $J_A x_s'(\tilde{p}) \neq 0$, and thus $x_s^A(p)$ is not constant over $\mathcal{P}$. □

We call (63) the *rank condition* for global invariance, since it can be tested numerically by checking that $\operatorname{rank}(P(p)|I(p)) = \operatorname{rank} I(p)$, where $I(p)$ is a matrix whose columns span the space $\mathcal{I}(p)$.

Next, we turn to the property of global concentration robustness. Note that concentration invariance usually depends on the exact values of parameters, e.g. reaction rate constants k_i, in the network model, i.e. a network may have global concentration invariance for one set of parameter values, but not for another set of parameter values. By the term *global concentration robustness*, we denote the property that a network has global concentration invariance independently of parameter values. As a consequence, if a network has global concentration robustness, all networks of the same structure, but potentially different parameter values, have global concentration invariance (and robustness).

Concerning the mass action part of the reaction rates, the interaction structure of the network is characterized by which stoichiometric coefficients are equal to zero. For the further steps, a similar notion is also needed for the generic part of the reaction rates, and is given by the following definition.

Definition 4. *Two vector valued functions $\Phi, \tilde{\Phi} : \mathbb{R}^q \to \mathbb{R}^k : y \mapsto \Phi(y), \tilde{\Phi}(y)$ are said to be structurally equivalent, if*

$$\frac{\partial \Phi_i}{\partial y_j} = 0 \quad \Leftrightarrow \quad \frac{\partial \tilde{\Phi}_i}{\partial y_j} = 0 \tag{65}$$

for all $i = 1, \ldots, k$ and $j = 1, \ldots, q$.

In the following, we will frequently consider a biochemical network with the same species vectors x and x^D as well as the same stoichiometric matrix N and link matrix L as the original network (42), but with potentially different reaction rates and a different vector of

total concentrations. This network is described by the equation

$$\begin{aligned} \dot{x} &= N\tilde{v}(x, x^D, p) \\ \tilde{x}^T(p) &= Lx + x^D, \end{aligned} \tag{66}$$

where $\tilde{v}$ has elements given by

$$\tilde{v}_i(x, x^D, p) = \tilde{\Phi}_i(x, x^D, p) \prod_{j=1}^{m} x_j^{a_{ij}} \prod_{j=1}^{n} (x_j^D)^{a_{ij}^D}. \tag{67}$$

The formal definition for global concentration robustness is as follows.

Definition 5. *The network* (42) *is said to have global concentration robustness, if all networks of the form* (66)*, where* $\tilde{\Phi}$ *and* $\tilde{x}^T$ *are structurally equivalent to* Φ *and* x^T*, respectively, have global concentration invariance.*

In what follows, we will derive a necessary and sufficient condition for global concentration robustness based on the rank condition established above for global concentration invariance.

First, the following technical definition for structural properties of a function Φ is introduced.

Definition 6. *Given a matrix* $\varphi \in \mathbb{R}^{k\times m}$ *and a function* $\Phi : \mathbb{R}^m \to \mathbb{R}^k$*, write* $\varphi \in \mathcal{S}(\frac{\partial\Phi}{\partial x})$ *if*

$$\varphi_{ij} = 0 \qquad \Leftrightarrow \qquad \frac{\partial \Phi_i}{\partial x_j} = 0 \tag{68}$$

for all i, j*.*

The result on global concentration robustness uses the two matrices M and P defined as follows, which depend on matrices $\varphi_x \in \mathbb{R}^{k\times m}$, $\varphi_{x^D} \in \mathbb{R}^{k\times n}$, $\varphi_{x^T} \in \mathbb{R}^n$, $\varphi_p \in \mathbb{R}^k$, and vectors $x \in \mathbb{R}^m_+$, $x^D \in \mathbb{R}^n_+$.

$$\begin{aligned} M(\varphi_x, \varphi_{x^D}, x^D, x) &= (\varphi_x + A - (\varphi_{x^D} + A^D)(\operatorname{dg} x^D)^{-1} L \operatorname{dg} x) J_M \\ P(\varphi_{x^D}, \varphi_{x^T}, \varphi_p) &= (\varphi_{x^D} + A^D)\varphi_{x^T} + \varphi_p \end{aligned} \tag{69}$$

Theorem 2. *The network* (42) *has global concentration robustness, if and only if for all* $\alpha \in \ker N$*,* $\varphi_x \in \mathcal{S}(\frac{\partial\Phi}{\partial x})$*,* $\varphi_{x^D} \in \mathcal{S}(\frac{\partial\Phi}{\partial x^D})$*,* $\varphi_{x^T} \in \mathcal{S}(\frac{\partial x^T}{\partial p})$*,* $\varphi_p \in \mathcal{S}(\frac{\partial\Phi}{\partial p})$*,* $x \in \mathbb{R}^m_+$*, and* $x^D \in \mathbb{R}^n_+$ *such that* $Lx + x^D > 0$

$$P(\varphi_{x^D}, \varphi_{x^T}, \varphi_p) \in \operatorname{im} M(\varphi_x, \varphi_{x^D}, x^D, x) + \ker(N \operatorname{dg} \alpha). \tag{70}$$

Proof. Sufficiency: Consider a network given by the equations (66) with reaction rates as in (67), where $\tilde{\Phi}$ and $\tilde{x}^T$ are structurally equivalent to Φ and x^T, respectively. Let $x_s(p)$ and $x_s^D(p)$ be steady state concentrations of this network and $\bar{v}(p)$ the corresponding steady state reaction rates. Define $\bar{\Phi}(p) = \tilde{\Phi}(x_s(p), x_s^D(p), p)$. Also define the following matrices:

$$\begin{aligned}
\varphi_p &= (\operatorname{dg}\bar{\Phi}(p))^{-1}\frac{\partial\tilde{\Phi}}{\partial p}(x_s(p), x_s^D(p), p)p \\
\varphi_x &= (\operatorname{dg}\bar{\Phi}(p))^{-1}\frac{\partial\tilde{\Phi}}{\partial x}(x_s(p), x_s^D(p), p)\operatorname{dg}x_s(p) \\
\varphi_{x^D} &= (\operatorname{dg}\bar{\Phi}(p))^{-1}\frac{\partial\tilde{\Phi}}{\partial x^D}(x_s(p), x_s^D(p), p)\operatorname{dg}x_s^D(p) \\
\varphi_{x^T} &= (\operatorname{dg}x_s^D(p))^{-1}\frac{\partial\tilde{x}^T}{\partial p}(p)p,
\end{aligned} \tag{71}$$

and note that $\varphi_p \in \mathcal{S}(\frac{\partial\Phi}{\partial p})$, $\varphi_x \in \mathcal{S}(\frac{\partial\Phi}{\partial x})$, $\varphi_{x^D} \in \mathcal{S}(\frac{\partial\Phi}{\partial x^D})$, and $\varphi_{x^T} \in \mathcal{S}(\frac{\partial x^T}{\partial p})$. The matrices P, Q, D, and M from (52) for the network (66) are computed as follows:

$$\begin{aligned}
P(p) &= \varphi_p + (A^D + \varphi_{x^D})\varphi_{x^T} \\
Q(p) &= A + \varphi_x \\
D(p) &= A^D + \varphi_{x^D} \\
M(p) &= \big(A + \varphi_x - (A^D + \varphi_{x^D})(\operatorname{dg}x_s^D(p))^{-1}L\operatorname{dg}x_s(p)\big)J_M.
\end{aligned} \tag{72}$$

The condition $P(\varphi_{x^D}, \varphi_{x^T}, \varphi_p) \in \operatorname{im}M(\varphi_x, \varphi_{x^D}, x^D, x) + \ker(N\operatorname{dg}\alpha)$ implies that

$$P(p) \in \operatorname{im}M(p) + \ker(N\operatorname{dg}\bar{v}(p)), \tag{73}$$

for all $p \in \mathcal{P}$, implying that the network (66) has global concentration invariance. Thus, by Definition 5, the network (42) has global concentration robustness.

Necessity: Assume that the condition $P(\varphi_{x^D}, \varphi_{x^T}, \varphi_p) \in \operatorname{im}M(\varphi_x, \varphi_{x^D}, x^D, x) + \ker(N\operatorname{dg}\alpha)$ is not satisfied for some $\alpha \in \ker N$, $\tilde{x} \in \mathbb{R}_+^m$, $\tilde{x}^D \in \mathbb{R}_+^n$ with $L\tilde{x} + \tilde{x}^D > 0$, and matrices $\varphi_x \in \mathcal{S}(\frac{\partial\Phi}{\partial x})$, $\varphi_{x^D} \in \mathcal{S}(\frac{\partial\Phi}{\partial x^D})$, $\varphi_{x^T} \in \mathcal{S}(\frac{\partial x^T}{\partial p})$, and $\varphi_p \in \mathcal{S}(\frac{\partial\Phi}{\partial p})$:

$$P(\varphi_{x^D}, \varphi_{x^T}, \varphi_p) \notin \operatorname{im}M(\varphi_x, \varphi_{x^D}, \tilde{x}^D, \tilde{x}) + \ker(N\operatorname{dg}\alpha), \tag{74}$$

Next, consider the network (66) with reaction rates as in (67), where $\tilde{\Phi}$ is chosen as

$$\tilde{\Phi}_i(x, x^D, p) = \tilde{k}_i \prod_{j=1}^{m} x_j^{(\varphi_x)_{ij}} \prod_{j=1}^{n} (x^D)^{(\varphi_{x^D})_{ij}} p^{(\varphi_p)_{ij}}. \tag{75}$$

Choose any $\tilde{p} \in \mathcal{P}$ and let

$$\tilde{k}_i = \alpha_i \prod_{j=1}^{m} \tilde{x}_j^{-a_{ij}-(\varphi_x)_{ij}} \prod_{j=1}^{n} (\tilde{x}^D)^{-a_{ij}^D-(\varphi_{x^D})_{ij}} \tilde{p}^{-(\varphi_p)_{ij}}, \tag{76}$$

for $i = 1, \ldots, k$. Furthermore, define

$$\bar{\varphi}_{x^T} = \operatorname{dg} \tilde{x}^D (\operatorname{dg}(L\tilde{x} + \tilde{x}^D))^{-1} \varphi_{x^T}, \tag{77}$$

and let

$$\tilde{x}_i^T(p) = (L\tilde{x} + \tilde{x}^D)_i \tilde{p}^{-(\bar{\varphi}_{x^T})_i} p^{(\bar{\varphi}_{x^T})_i}, \tag{78}$$

for $i = 1, \ldots, n$. Note that $\tilde{\Phi}$ and Φ as well as $\tilde{x}^T$ and x^T are structurally equivalent due to the structural constraints on the matrices φ_x, φ_{x^T}, φ_p, and φ_{x^T}. With the definition of $\tilde{k}_i$ in (76), $\tilde{v}(\tilde{x}, \tilde{x}^D, \tilde{p}) = \alpha$. Then, since $\alpha \in \ker N$, and also $\tilde{x}^T(\tilde{p}) = L\tilde{x} + \tilde{x}^D$, the network (66) with $\tilde{\Phi}$, $\tilde{x}^T$ as just defined has a steady state given by

$$\begin{aligned} x_s(\tilde{p}) &= \tilde{x} \\ x_s^D(\tilde{p}) &= \tilde{x}^D. \end{aligned} \tag{79}$$

and steady state reaction rates

$$\bar{v}(\tilde{p}) = \alpha. \tag{80}$$

It remains to show that (66) does not have local concentration robustness at the perturbation $\tilde{p}$. The matrices P and M defined in (52) are computed for the network (66) as follows:

$$\begin{aligned} P(\tilde{p}) &= \varphi_p + (A^D + \varphi_{x^D})\varphi_{x^T} \\ M(\tilde{p}) &= (A + \varphi_x - (A^D + \varphi_{x^D})(\operatorname{dg} \tilde{x}^D)^{-1} L \operatorname{dg} \tilde{x}) J_M \end{aligned} \tag{81}$$

Thus, from condition (74), we find that

$$P(\tilde{p}) \notin \operatorname{im} M(\tilde{p}) + \ker(N \operatorname{dg} \bar{v}(\tilde{p})), \tag{82}$$

implying that the network (66) does not have local concentration robustness at $\tilde{p}$. Thus, by Definition 5, the network (42) does not have global concentration robustness.

□

Note that the condition (70) can again be rephrased as the rank condition $\operatorname{rank}(P|I) = \operatorname{rank} I$, where I is a matrix whose columns span the space $\operatorname{im} M + \ker(N \operatorname{dg} \alpha)$.

V. ROBUSTNESS OF THE *ESCHERICHIA COLI* CHEMOTAXIS PATHWAY

From the stationary equations of the reaction rates, given in Materials and Methods of the main text, we determine the logarithmic expansion coefficients for the state variables $\boldsymbol{M}$ and the perturbations $\boldsymbol{P}$. In the following only nonzero coefficients are considered and the results are summarized in Table I.

We make use of Kronecker's delta, defined by $\delta_{ij} = 1$ for $i = j$ and zero otherwise. If perturbations act on individual components we arrive at

$$\frac{\partial \ln v_i}{\partial \ln R^T} = \delta_{i1} \quad (83)$$

$$\frac{\partial \ln v_i}{\partial \ln B^T} = \delta_{i3} \quad (84)$$

$$\frac{\partial \ln v_i}{\partial \ln Y^T} = -\delta_{i3} \quad (85)$$

$$\frac{\partial \ln v_i}{\partial \ln Z^T} = \delta_{i6} \quad (86)$$

$$\frac{\partial \ln v_i}{\partial \ln A^T} = (\delta_{i3} + \delta_{i5}) \underbrace{\frac{\partial \ln A^c}{\partial \ln A^T}}_{\beta_1} \quad (87)$$

$$\frac{\partial \ln v_i}{\partial \ln W^T} = (\delta_{i3} + \delta_{i5}) \underbrace{\frac{\partial \ln A^c}{\partial \ln W^T}}_{\beta_2} \quad (88)$$

$$\frac{\partial \ln v_i}{\partial \ln T} = (\delta_{i3} + \delta_{i5}) \underbrace{\frac{\partial \ln A^c}{\partial \ln T}}_{\beta_3} - \delta_{i1} - \delta_{i2} \quad (89)$$

$$\frac{\partial \ln v_i}{\partial \ln [ATP]} = (\delta_{i3} + \delta_{i5}) \underbrace{\frac{\partial \ln k_A(\mathrm{ATP})}{\partial \ln [ATP]}}_{\delta} \quad (90)$$

$$\frac{\partial \ln v_i}{\partial \ln L} = (\delta_{i3} + \delta_{i5}) \underbrace{\frac{\partial \ln P^s}{\partial \ln L}}_{\alpha_2} \quad (91)$$

$$\frac{\partial \ln v_i}{\partial \ln \mathrm{mRNA}_{mocha}} = \frac{\partial \ln v_i}{\partial \ln R^T} + \frac{\partial \ln v_i}{\partial \ln B^T} + \frac{\partial \ln v_i}{\partial \ln Y^T} + \frac{\partial \ln v_i}{\partial \ln Z^T} \quad (92)$$

which results in the matrix shown in Fig. 2 of the main text.

The non-zero entries of $\boldsymbol{M}$ are given by

$$\frac{\partial \ln v_i}{\partial \ln Bp} = \delta_{i2} + \delta_{i4} \quad (93)$$

$$\frac{\partial \ln v_i}{\partial \ln m} = (\delta_{i3} + \delta_{i5}) \underbrace{\frac{\partial \ln P^s}{\partial \ln m}}_{\alpha_1} . \quad (94)$$

TABLE I: The logarithmic expansion coefficients reflecting the matrix $(\boldsymbol{A}|\boldsymbol{M}|\boldsymbol{P}^{(1)}|\boldsymbol{P}^{(2)})$. Note that the rate equations contain nonlinear functions $k_A = k_A(\text{ATP})$, $P^s = P^s(m, L)$ and $A^c = A^c(A^T, W^T, T)$. Lowercase Greek letters denote the logarithmic expansion coefficients that arise from (unspecified) nonlinear dependencies. We emphasize that, though in this case the precise functional dependencies are known, our framework does not require to specify the exact functional form of the dependencies.

	Y_p	m	B_p	L	A^T	W^T	T	ATP	R^T	B^T	Y^T	Z^T
$v_1 = k_R\, R^T/T$	0	0	0	0	0	0	-1	0	1	0	0	0
$v_2 = k_B\, B_p/T$	0	0	1	0	0	0	-1	0	0	0	0	0
$v_3 = k_A\, A^c\, P^s\, \dfrac{K_Y}{K_B} \dfrac{B^T}{Y^T}$	0	α_1	0	α_2	β_1	β_2	β_3	δ	0	1	-1	0
$v_4 = \gamma_B\, B_p$	0	0	1	0	0	0	0	0	0	0	0	0
$v_5 = k_A\, A^c\, P^s$	0	α_1	0	α_2	β_1	β_2	β_3	δ	0	0	0	0
$v_6 = k_Z\, Z^T \dfrac{Y_p}{K_Z + Y_p}$	η	0	0	0	0	0	0	0	0	0	0	1

As demonstrated in the main text, the specific topological organization, with kinetic dependencies summarized in Table I, allows the output of the chemotactic pathway to be robust against diverse perturbations that would otherwise impede the functionality of the network. As can easily be ascertained in Table I each perturbation corresponding to the columns of $\boldsymbol{P}^{(1)}$ is an element of the invariant perturbation space. In contrast to this, perturbations corresponding to the columns of $\boldsymbol{P}^{(2)}$ are not within the invariant perturbation space, hence the pathway is not robust against fluctuations in these components. However, the organization of the pathway ensures that the pathway is indeed robust against concerted fluctuations in these components. See main text for details.

We emphasize that the uncovered design principle of the chemotaxis pathway is in contrast to the more straightforward possibility to utilize an extensive cellular machinery to 'fine-tune' quantities that appear as parameters in the equations, such as protein concentrations or ATP availability. We further note that the robustness requires some logarithmic derivatives to attain specific values, as encoded for example by mass-action kinetics, while other functional dependencies may remain unspecified.

VI. MATERIALS AND METHODS

A. Bacterial strains and plasmids

VS104 [Δ(cheYcheZ)] and LL4 [Δ(cheYcheZ)ΔflgM] strains used in this study were derived from a wild-type chemotaxis strain RP437 using pAMPts homologous recombination system of allele exchange as described before [11]. Plasmid pVS88 encodes CheY-YFP and CheZ-CFP fusion proteins transcribed as one bicistronic mRNA from the pTrc promoter inducible by isopropyl β-D-thiogalactoside (IPTG) [13].

B. Growth conditions

All strains were grown under standard chemotaxis conditions [11, 13] at 34C in a rotary shaker to mid-exponential phase (OD600 ≈ 0.48) in tryptone broth (TB) supplemented with 100μg/ml ampicillin and indicated amounts of IPTG.

C. FRET measurements

Cell preparation, FRET measurements and evaluation of FRET data were performed as described previously [12, 14] on a custom-modified Zeiss Axiovert 200 microscope.

D. Quantification of gene expression

Expression of fluorescent reporter proteins in individual cells was quantified as described before [5] using fluorescence imaging on an AxioImager fluorescence microscope equipped with an ORCA AG CCD camera (Hamamatsu).

E. Note on Figure 4B

Measurements of kinase activity upon stimulation by CheZ-CFP/CheY-YFP FRET in a CheY/CheZ deleted strain (VS104; black line) and a strain with additional deletion of the anti-sigma factor flgM (LL4; red line), where the latter results in an approximately seven fold upregulated transcriptional activity of the pathway proteins, including mocha, meche

operons and chemoreceptors. As the FRET pair is expressed from plasmid, a shift in the adapted kinase activity occurs upon upregulation of pathway proteins. This shift is correct by employing measurements of the flagellar rotation bias in a wild type strain and a flgM deleted strain (CheY and CheZ native), where it has been shown that in both strains the adapted kinase activities are equal [5]. The resulting rescaled kinase activity is shown in Fig. 4B.

VII. FURTHER APPLICATIONS

A. Two-component systems and implications for synthetic biology

One of the merits of our approach is to guide the design of perfectly robust signaling circuits – with important implications for synthetic biology. To exemplify the construction of robust signalling networks, we briefly consider instances of two-component signal transduction systems. Bacterial two-component systems typically consist of a membrane-bound sensor kinase that senses a specific stimulus and a cognate response regulator that modulates the signal response. The robustness of individual bacterial two-component system with respect to concentration fluctuations was investigated previously [2, 9].

Recently, Skerker et al. [10] described a method that allows for the rational rewiring of the specificity of two-component systems. Such rational design of the output responses of two-component systems is a major step forward in the design of protein-based synthetic pathways, with exciting potential applications in synthetic biology and biotechnology [4]. However, the rational design of two-component systems will also necessitate to engineer robustness of the rewired pathways with respect to possible detrimental fluctuations – taking into account that synthetic circuits are not a product of evolution. In this respect, of particular importance are fluctuations in compound concentrations that arise from stochastic variations in transcription and translation, as well as from other sources, such as variations in division. In fact, it seems highly desirable to implement any altered topology such that the expression of the individual proteins has no effect on the (rationally designed) input-output relationship of the network. In this case, the only requirement for the method of Skerker et al. [10] to generate perfectly robustness networks is a sufficiently high expression of any of the involved proteins – without the need to fine-tune any of the precise expression levels.

Our framework is able to straightforwardly account for the mechanisms of robustness of such (networks of) two-component systems. In Fig. 1 two variants of a prototypical two-component system are shown, each consisting only of three reactions: the autophosphorylation of a sensor histidine kinase (H), the transfer of the phosphoryl group to a response regulator (R), and the subsequent dephosphorylation of R. Neglecting complex formation (but see below for the full solution), the system is described by the two differential equations

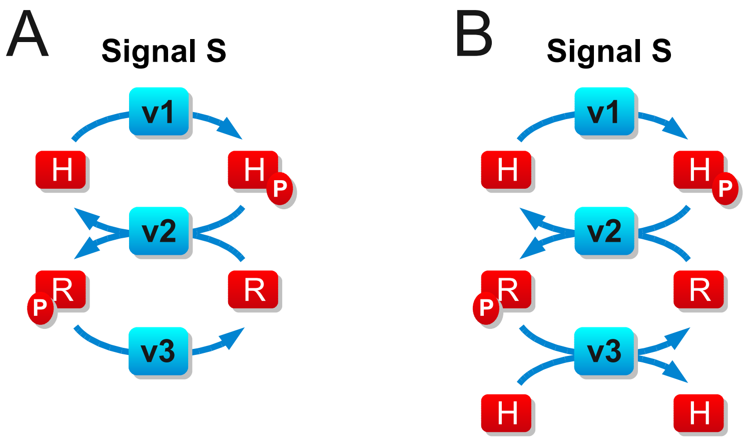

FIG. 1: Robustness of two-component systems. We consider three simplified reactions, autophosphorylation of a sensor histidine kinase (H), transfer of the phosphoryl group to a response regulator (R), and dephosphorylation of R. (**A**) The prototypical two-component system. (**B**) A modified topology with a bifunctional histidine kinase, such that the unphosphorylated kinase acts as a phosphatase for the response regulator.

for the independent state variables, H_P and R_P,

$$\frac{d}{dt}\begin{pmatrix} H_P \\ R_P \end{pmatrix} = \underbrace{\begin{pmatrix} 1 & -1 & 0 \\ 0 & 1 & -1 \end{pmatrix}}_{\boldsymbol{N}} \cdot \begin{pmatrix} \nu_1 \\ \nu_2 \\ \nu_3 \end{pmatrix} \tag{95}$$

and the mass conservation relationship

$$\begin{pmatrix} H^T \\ R^T \end{pmatrix} = \underbrace{\begin{pmatrix} 1 & 0 \\ 0 & 1 \end{pmatrix}}_{\boldsymbol{L}} \begin{pmatrix} H_P \\ R_P \end{pmatrix} + \begin{pmatrix} H \\ R \end{pmatrix} \quad . \tag{96}$$

Both topologies only differ in kinetic dependencies of the rate equations, specifically

$$\boldsymbol{\nu_A} = \begin{pmatrix} k_1 \cdot S \cdot H \\ k_2 \cdot H_P \cdot R \\ k_3 \cdot R_P \end{pmatrix} \qquad \text{and} \qquad \boldsymbol{\nu_B} = \begin{pmatrix} k_1 \cdot S \cdot H \\ k_2 \cdot H_P \cdot R \\ k_3 \cdot R_P \cdot H \end{pmatrix} \tag{97}$$

for the systems shown in Figs. 1A and 1B, respectively. The right nullspace of the scaled stoichiometry is identical for both systems and can be represented by a matrix $\boldsymbol{K}$ that solely consist of the **1**-vector (here the column has already been normalized to remove dependencies

on the stationary flux distribution).

We aim to test for invariance of the pathway output – the phosphorylated response regulator (R_P) – with respect to variations in total component concentrations H^T and R^T. We proceed as described in Sections II.B and II.C.

We start with the topology shown in Fig. 1A. In this case, the matrices of logarithmic partial derivatives are given as

$$\boldsymbol{M^D} = \left(\begin{array}{c|c} & H_p \\ \hline v_1 & 0 \\ v_2 & 1 \\ v_3 & 0 \end{array}\right) \quad \text{and} \quad \boldsymbol{D} = \left(\begin{array}{c|cc} & H & R \\ \hline v_1 & 1 & 0 \\ v_2 & 0 & 1 \\ v_3 & 0 & 0 \end{array}\right), \tag{98}$$

resulting in a matrix $\boldsymbol{M}$ defined as (see Section II.B)

$$\boldsymbol{M} = \left(\begin{array}{c|c} & H_p \\ \hline v_1 & 0 \\ v_2 & 1 \\ v_3 & 0 \end{array}\right) - \left(\begin{array}{c|cc} & H & R \\ \hline v_1 & 1 & 0 \\ v_2 & 0 & 1 \\ v_3 & 0 & 0 \end{array}\right) \cdot \underbrace{\begin{pmatrix} -\alpha \\ 0 \end{pmatrix}}_{\boldsymbol{L''}} = \left(\begin{array}{c|c} & H_p \\ \hline v_1 & \alpha \\ v_2 & 1 \\ v_3 & 0 \end{array}\right), \tag{99}$$

where $\alpha = -H_p/H$. The perturbation vectors with respect to the total concentrations H^T and R^T are given as

$$\boldsymbol{P_H} = \left(\begin{array}{c|c} & H^T \\ \hline v_1 & \gamma_H \\ v_2 & 0 \\ v_3 & 0 \end{array}\right) \quad \text{and} \quad \boldsymbol{P_R} = \left(\begin{array}{c|c} & R^T \\ \hline v_1 & 0 \\ v_2 & \gamma_R \\ v_3 & 0 \end{array}\right), \tag{100}$$

with $\gamma_H := \partial \ln \nu_1 / \partial \ln H^T$ and $\gamma_R := \partial \ln \nu_2 / \partial \ln R^T$.

The rank condition for global concentration robustness with respect to the total concentration of the histidine kinase (H^T) therefore reads

$$\text{rank} \left(\begin{array}{c|cc} \gamma_H & \alpha & 1 \\ 0 & 1 & 1 \\ 0 & 0 & 1 \end{array}\right) \stackrel{?}{=} \text{rank} \begin{pmatrix} \alpha & 1 \\ 1 & 1 \\ 0 & 1 \end{pmatrix}. \tag{101}$$

Obviously, the equation cannot be fulfilled for arbitrary values of γ_H and α, hence the system shown in Fig. 1A does *not* exhibit global concentration robustness with respect to the

total concentration of the histidine kinase (H^T).

The same conclusion can be reached for the total concentration of the response regulator (R^T). The rank condition for global concentration robustness reads

$$\operatorname{rank}\left(\begin{array}{c|cc} 0 & \alpha & 1 \\ \gamma_R & 1 & 1 \\ 0 & 0 & 1 \end{array}\right) \stackrel{?}{=} \operatorname{rank}\left(\begin{array}{cc} \alpha & 1 \\ 1 & 1 \\ 0 & 1 \end{array}\right) . \tag{102}$$

Again, the equation cannot be fulfilled for arbitrary values of γ_R and α, hence global concentration robustness is not achieved.

A different scenario is shown in Fig. 1B. Here, a bifunctional histidine kinase implies that the unphosphorylated kinase acts as a phosphorylase for the response regulator. Repeating the calculations shown above, we obtain

$$\boldsymbol{M^D} = \left(\begin{array}{c|c} & H_p \\ \hline v_1 & 0 \\ v_2 & 1 \\ v_3 & 0 \end{array}\right) \quad \text{and} \quad \boldsymbol{D} = \left(\begin{array}{c|cc} & H & R \\ \hline v_1 & 1 & 0 \\ v_2 & 0 & 1 \\ v_3 & 1 & 0 \end{array}\right) , \tag{103}$$

resulting in,

$$\boldsymbol{M} = \left(\begin{array}{c|c} & H_p \\ \hline v_1 & \alpha \\ v_2 & 1 \\ v_3 & \alpha \end{array}\right) \tag{104}$$

and

$$\boldsymbol{P_H} = \left(\begin{array}{c|c} & H^T \\ \hline v_1 & \gamma_H \\ v_2 & 0 \\ v_3 & \gamma_H \end{array}\right) \quad \text{and} \quad \boldsymbol{P_R} = \left(\begin{array}{c|c} & R^T \\ \hline v_1 & 0 \\ v_2 & \gamma_R \\ v_3 & 0 \end{array}\right) , \tag{105}$$

with definitions given above. We emphasize that indeed $\gamma_H := \partial \ln \nu_1 / \partial \ln H^T = \partial \ln \nu_3 / \partial \ln H^T$, with

$$\frac{\partial \ln \nu_1}{\partial \ln H^T} = \frac{H^T}{\nu_1}\frac{\partial\,(k_1 S H)}{\partial H^T} = \frac{\partial \ln H}{\partial \ln H^T} \quad \text{and} \quad \frac{\partial \ln \nu_3}{\partial \ln H^T} = \frac{H^T}{\nu_3}\frac{\partial\,(k_3 R_p H)}{\partial H^T} = \frac{\partial \ln H}{\partial \ln H^T} . \tag{106}$$

Testing the rank condition for both perturbations, reveals that

$$\text{rank}\begin{pmatrix} \gamma_H & \alpha & 1 \\ 0 & 1 & 1 \\ \gamma_H & \alpha & 1 \end{pmatrix} = \text{rank}\begin{pmatrix} \alpha & 1 \\ 1 & 1 \\ \alpha & 1 \end{pmatrix} \tag{107}$$

and

$$\text{rank}\begin{pmatrix} 0 & \alpha & 1 \\ \gamma_R & 1 & 1 \\ 0 & \alpha & 1 \end{pmatrix} = \text{rank}\begin{pmatrix} \alpha & 1 \\ 1 & 1 \\ \alpha & 1 \end{pmatrix} \quad , \tag{108}$$

thus both conditions are fulfilled for any non-zero values of γ_H, γ_R, and α. The system shown in Fig. 1B indeed exhibits perfect concentration robustness of the output variable R_p with respect to variations in both total concentrations H^T and R^T. We note that within this example the bifunctionality of the histidine kinase is crucial to achieve robustness of the pathway output – a mechanism that is functionally similar to the concerted expression of proteins adjacent on an operon observed for the *E. coli* chemotaxis pathway.

Elaborating on the simple system discussed above, our framework is also straightforwardly applicable to the full system, including explicit complex formation. We consider the three processes

$$H \rightarrow H_P \tag{109}$$

$$H_P + R \leftrightarrow [H_P R] \rightarrow R_P + H \tag{110}$$

$$R_P + H \leftrightarrow [R_P H] \rightarrow R + H \quad . \tag{111}$$

corresponding to system of 6 variables and 7 reaction rates. The system of differential equations is given as

$$\frac{d}{dt}\begin{pmatrix} H_P \\ R_P \\ [H_P R] \\ [R_P H] \end{pmatrix} = \underbrace{\begin{pmatrix} 1 & -1 & 1 & 0 & 0 & 0 & 0 \\ 0 & 0 & 0 & 1 & -1 & 1 & 0 \\ 0 & 1 & -1 & -1 & 0 & 0 & 0 \\ 0 & 0 & 0 & 0 & 1 & -1 & -1 \end{pmatrix}}_{\boldsymbol{N}} \cdot \underbrace{\begin{pmatrix} k_1 \cdot H \\ k_2 \cdot H_P \cdot R \\ k_3 \cdot [H_P R] \\ k_4 \cdot [H_P R] \\ k_5 \cdot R_P \cdot H \\ k_6 \cdot [R_P H] \\ k_7 \cdot [R_P H] \end{pmatrix}}_{\boldsymbol{v}} \quad , \tag{112}$$

and supplemented by the mass conservation relationship

$$\begin{pmatrix} H^T \\ R^T \end{pmatrix} = \underbrace{\begin{pmatrix} 1 & 0 & 1 & 1 \\ 0 & 1 & 1 & 1 \end{pmatrix}}_{\boldsymbol{L}} \begin{pmatrix} H_P \\ R_P \\ [H_P R] \\ [R_P H] \end{pmatrix} + \begin{pmatrix} H \\ R \end{pmatrix} . \tag{113}$$

Here the concentrations of unphosphorylated components, R and H, are chosen as dependent variables. We note that the choice of dependent variables is not unique. Alternative choices lead to identical results, provided that the dependent variables are chosen such that the matrix $\boldsymbol{M^D}$ is of maximal possible rank.

To test for robustness of the pathway output, we first evaluate the nullspace of the stoichiometry,

$$\boldsymbol{K}^N = \begin{pmatrix} 1 & 0 & 0 \\ 1 & 1 & 0 \\ 0 & 1 & 0 \\ 1 & 0 & 0 \\ 1 & 0 & 1 \\ 0 & 0 & 1 \\ 1 & 0 & 0 \end{pmatrix} \quad \rightarrow \quad \boldsymbol{K'} = \begin{pmatrix} 1 \\ 1 \\ 1 \\ 1 \\ 1 \\ 1 \\ 1 \end{pmatrix} , \tag{114}$$

where the representation has already been normalized and dependencies on the stationary flux distribution were already removed. The logarithmic expansion coefficients for the state variables $\boldsymbol{M^D}$ are summarized in Table II. Instead of computing the expression for $\boldsymbol{M} = \boldsymbol{M^D} - \boldsymbol{D} \cdot \boldsymbol{L''}$, we utilize a direct approach to judge output robustness of the network. Since we are only interested in the vector space spanned by the matrices $\boldsymbol{M}$ and $\boldsymbol{K'}$, and not in a particular representation, we note that the expression for $\boldsymbol{M}$ is not required under the condition $\text{rank}(\boldsymbol{D}|\boldsymbol{M^D}|\boldsymbol{K'}) = \text{rank}(\boldsymbol{M^D}|\boldsymbol{K'})$. As can be verified in Table II this condition indeed holds for the two-component system. Furthermore, the pertubations $\boldsymbol{P_T}$ with respect to total concentrations can be expressed in terms of the partial logarithmic derivatives with respect to the dependent variables. Since $\text{rank}(\boldsymbol{D}|\boldsymbol{M^D}|\boldsymbol{K'}) = \text{rank}(\boldsymbol{M^D}|\boldsymbol{K'})$, obviously also $\text{rank}(\boldsymbol{P_T}|\boldsymbol{M^D}|\boldsymbol{K'}) = \text{rank}(\boldsymbol{M^D}|\boldsymbol{K'})$. Hence the pathway exhibits perfect robustness against variations in the total concentrations R^T and H^T.

This result can be generalized to a generic strategy towards perfect output robustness for engineered protein networks. Utilizing rewiring of substrate specificity [10], in addition

TABLE II: The logarithmic expansion coefficients with respect to the dependent and independent state variables, $\boldsymbol{D}$ and $\boldsymbol{M^D}$, respectively, along with the nullspace $\boldsymbol{K'}$.

	$\boldsymbol{D}$			$\boldsymbol{M^D}$			$\boldsymbol{K'}$
	R_p	R	H	$[H_P R]$	$[R_P H]$	H_P	
$v_1 = k_1 \cdot H$	0	0	1	0	0	0	1
$v_2 = k_2 \cdot H_P \cdot R$	0	1	0	0	0	1	1
$v_3 = k_3 \cdot [H_P R]$	0	0	0	1	0	0	1
$v_4 = k_4 \cdot [H_P R]$	0	0	0	1	0	0	1
$v_5 = k_5 \cdot R_P \cdot H$	1	0	1	0	0	0	1
$v_6 = k_6 \cdot [R_P H]$	0	0	0	0	1	0	1
$v_7 = k_7 \cdot [R_P H]$	0	0	0	0	1	0	1

to implement the desired functionality, the interactions should be rewired such that the logarithmic expansion coefficients for the dependent state variables $\boldsymbol{D}$ are linearly dependent on the logarithmic expansion coefficients for the independent state variables $\boldsymbol{M^D}$ and the largest parameter independent representation of the nullspace $\boldsymbol{K'}$. Specifically, we require

$$\mathrm{rank}(\boldsymbol{D}|\boldsymbol{M^D}|\boldsymbol{K'}) = \mathrm{rank}(\boldsymbol{M^D}|\boldsymbol{K'}) \ , \tag{115}$$

to ensure global concentration robustness against all total concentrations within a signaling network. This simple condition allows to establish whether a rewired network will exhibit the desired functionality without the need to fine-tune expression levels. Our framework is able to guide the necessary network extensions to guarantee robust network functionality.

B. Conservation relationships and robustness of mass-action systems

A particular application of our framework relates to global concentration robustness of mass-action systems with respect to total conserved concentrations, as recently also discussed elsewhere [8]. Our framework allows to derive a simple principle that allows to judge for global concentration robustness and is able to guide the necessary network extensions to design perfectly robust networks. We consider a system as described in Eq. (7) in Sec-

tion II B,

$$\dot{\boldsymbol{x}} = \boldsymbol{N} \cdot \boldsymbol{v}\left(\boldsymbol{x}, \boldsymbol{x}^D\right) \tag{116}$$

$$\boldsymbol{x}^T = \boldsymbol{L} \cdot \boldsymbol{x} + \boldsymbol{x}^D \quad , \tag{117}$$

and aim to test for robustness of the output variables $\boldsymbol{x^A}$ with respect to perturbation in the total concentration of each molecular component $\boldsymbol{x}^T = (x_1^T, x_2^T, ..., x_n^T)$. For simplicity, we assume that all rate equations are given by generalized mass-action (GMA) kinetics,

$$v_i(\boldsymbol{x}) = k_i \prod_{j=1}^{n} x_j^{\alpha_{ij}}. \tag{118}$$

Consequently, the partial logarithmic derivatives with respect to dependent and independent variables, the elements of the matrices $\boldsymbol{D}$ and $\boldsymbol{M^D}$, are constant values. Under the special condition

$$\text{rank}(\boldsymbol{D}|\boldsymbol{M^D}|\boldsymbol{K'}) = \text{rank}(\boldsymbol{M^D}|\boldsymbol{K'}) \quad , \tag{119}$$

where $\boldsymbol{K'}$ denotes a largest parameter-independent representation of the nullspace, the system exhibits global concentration robustness with respect to perturbations in $\boldsymbol{x}^T$. The reason is that any perturbation in total concentrations can be represented by a perturbation in the dependent variables $\boldsymbol{x^D}$. Since $\boldsymbol{D}$ is already an element of the invariant perturbation space, due to the condition Eq. (119), any such perturbation is necessarily also an element of the invariant space, hence global concentration robustness with respect to total conserved concentrations is guaranteed.

As compared to alternative methods [8], our approach has the advantages that it is (i) conceptually considerably simpler, (ii) numerically straightforward to test by standard methods of linear algebra (rank conditions), and (iii) straightforwardly guiding modification of the matrix $\boldsymbol{D}$ to ensure perfect concentration robustness.

C. Complex perturbations and temperature compensation

One of the merits of our approach is that it is not restricted to a particular type of perturbation, but is applicable to a wide variety of detrimental influence that potentially impede network functionality. While our focus is mainly on variations in native expression levels – as one of the dominant sources of variability in living cells – our framework also

accounts for any other perturbation that can be expressed in terms of the logarithmic expansion coefficient of the rate equations.

Relevant applications include the retroactivity of signaling circuits [15] as well as detrimental pathway crosstalk [1, 6]. Both issues also relate to scenarios where signaling pathways utilize common resources, such as ATP to provide energy. Within our framework, any such possibly detrimental influence can be considered as a perturbation – allowing the identification or construction of an appropriate topology that compensates for the corresponding perturbation.

A particular intriguing example of a complex perturbation is given by variations in temperature. A change in temperature usually affects all rate constants simultaneously – making the prediction of perfectly robust topologies a difficult task [7]. In the simplest case, we may assume that each reaction rate follows the Arrhenius equation, that is, the temperature dependence of a reaction rate v_i can be described by a multiplicative factor

$$k_i = A_i \exp\left(-\frac{E_i}{RT}\right) \quad , \tag{120}$$

where E_i denotes the activation energy for the ith reaction, A_i a proportionality constant, R the gas constant, and T the absolute temperature in Kelvin). Here, the activation energy is a constant for each reaction that does not further dependent on the temperature or the stationary state. In this case, we can straightforwardly construct the perturbation vector $\boldsymbol{P_T}$ with respect to changes in temperature, with elements

$$\frac{\partial \ln v_i}{\partial \ln T} = \frac{\partial \ln k_i}{\partial \ln T} = \frac{E_i}{RT} \quad . \tag{121}$$

Specifically, each element of $\boldsymbol{P_T}$ explicitly depends on the temperature. However, the *direction* of the vector $\boldsymbol{P_T}$, with

$$\boldsymbol{P_T} = \frac{1}{RT}\begin{pmatrix} E_1 \\ E_2 \\ \vdots \\ E_r \end{pmatrix} \tag{122}$$

for a system consisting of r reaction rates, does only depend on the (constant) activation energies of the reaction. Hence, using our condition for output robustness it is straightforward to judge global temperature compensation of a biochemical network.

We note that in practise a straightforward application of the Arrhenius equation is often not

appropriate. In this case, for arbitrary dependencies $k_i(T)$ our framework is still applicable – just as for any other complex perturbation that acts on many reaction rates simultaneously.

D. Robustness of bi- and multistable systems

As yet, the focus of our approach has been networks that exhibit a globally stable stationary state, characterized by $\boldsymbol{x^s}$ and $\boldsymbol{\nu^s}$, for all parameters. However, many signaling networks exhibit bi -or multistable dynamics, giving rise to two or more stationary states. As a particular merit, our approach is still applicable in these situations – without requiring substantial modifications. In particular, global uniqueness of the stationary state was not a necessary precondition to derive the requirement for global robustness, hence the condition for global concentration robustness applies to any locally stable state that fulfills the steady state condition.

However, our framework does rely on invertibility of the Jacobian matrix to ensure that a gradual change of intermediate variables may not affect the set of designated output variables. This does not hold in a situation in which the system, under the action of a perturbation, undergoes a bifurcation that results in a non-invertible Jacobian (as, for example, a saddle-node bifurcation). With respect to the application on multistable systems, we therefore have to introduce the *additional constraint* that all perturbations must be such that the transient response in systems variables after the perturbation remains within the *basin of attraction* of the respective state. Once the perturbation is sufficiently strong to allow the system to cross the attractor boundary, the robustness of the state is lost. However, in this case the system usually adopts another stationary state – which again exhibits perfect concentration robustness with respect to perturbations of large magnitude. In this sense, we are in the favorable situation that our framework allows to construct robust bi-or multistable systems that are still capable to switch between states. Also, since we are mainly concerned with slow perturbations with respect to the intrinsic timescales of the system, the robustness of each stationary state is maintained even for perturbations of comparatively large magnitude – provided the variables remain within the basin of attraction of the respective state.

As a guideline for the construction of robust bi- or multistable systems, we further note that such systems usually involve strong nonlinearities. Here, it is of considerable advantage

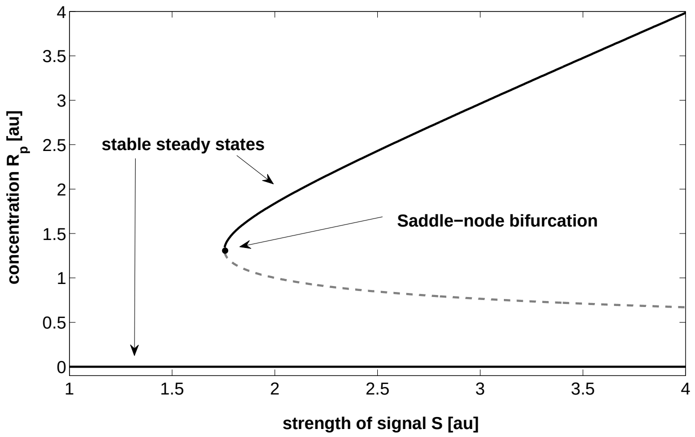

FIG. 2: A bistable system based on a generic two-component architecture that exhibits perfect concentration robustness. Shown is the stationary state of the active response regulator R_p as a function of the signal strength S. For sufficiently large S a non-zero solution exists, in addition to the solution $R_p = 0$. All stationary states, including the separatrix (grey dashed line), are invariant with respect to changes in total conserved proteins H^T and R^T. Parameters are $k_1 = k_3 = 1$, $K_a = 1$, $n = 4$, and $H^T = R^T = 5$, each given in arbitrary units (au).

to utilize a robust ("output") variable within the feedback mechanism. In this way, the requirement to fine-tune a highly nonlinear feedback is circumvented.

We illustrate the design of a multistable robust system using a simple example based on a generic two-component system. We again consider the system of differential equations discussed above

$$\frac{d}{dt}\begin{pmatrix} H_P \\ R_P \end{pmatrix} = \underbrace{\begin{pmatrix} 1 & -1 & 0 \\ 0 & 1 & -1 \end{pmatrix}}_{\boldsymbol{N}} \cdot \begin{pmatrix} \nu_1 \\ \nu_2 \\ \nu_3 \end{pmatrix} \quad . \tag{123}$$

The vector of kinetic rate equations $\boldsymbol{\nu}$ is analogous to the robust topology shown in Fig. 1B.

$$\boldsymbol{\nu} = \begin{pmatrix} k_1 \cdot S \cdot H \cdot f(R_p) \\ k_2 \cdot H_P \cdot R \\ k_3 \cdot R_P \cdot H \end{pmatrix} \quad . \tag{124}$$

As the only modification, we assume that the active response regulator R_p is capable to sensitize the receptor, thereby enhancing the phosphorylation of H. This modification does not impede the robustness of the system with respect to changes in the total conserved

protein concentrations. In particular, the derivative towards the variable R_p does not enter the invariant perturbation space or its construction, hence the invariant perturbation space is identical to the scenario discussed above.

Solving for the steady state of the system using the relationship $\nu_1 = \nu_3$ at steady state, we obtain

$$R_p - \frac{k_1}{k_3} \cdot S \cdot f(R_p) = 0 \quad . \tag{125}$$

With a particular choice of $f(R_p)$, for example,

$$f(R_p) = \frac{{R_p}^n}{{K_a}^n + {R_p}^n} \quad , \tag{126}$$

and $n = 4$ we obtain

$$R_p^5 - \frac{k_1}{k_3} \cdot S \cdot {R_p}^4 + {K_a}^n R_p = 0 \quad , \tag{127}$$

and the system indeed exhibits bistable dynamics that is independent of the total concentrations H^T and R^T. A bifurcation diagram is shown in Fig. 2.

The system allows for several conclusions: (i) Concentration robustness holds for any stationary state of the system, including unstable states. (ii) Strong nonlinearities should be confined to robust variables. As these variables do not enter the construction of the invariant perturbation space, the need for fine-tuning the respective parameters is circumvented. (iii) Robustness of the system is lost at the bifurcation. In this case, the state looses stability and adopts another state, which again exhibits non-local robustness. Hence, robustness is restricted to perturbations within the respective basin of attraction. (iv) While these conclusions may be obvious for the simple example discussed above, our reasoning likewise applies to systems of large size and is amendable to an algorithmic solution.

VIII. MISCELLANEA

A. Limitations of our approach

As the reader may have noticed, a physical network that is robust in the sense $rank(\boldsymbol{P}|\boldsymbol{M}|\boldsymbol{K}) = rank(\boldsymbol{M}|\boldsymbol{K})$, with $rank(\boldsymbol{P}|\boldsymbol{K}) \neq rank(\boldsymbol{K})$, cannot be robust to arbitrary large perturbation strength. The reason is the perturbation induces changes in $\Delta\boldsymbol{x}^M$. As the intermediate state variables, $\Delta\boldsymbol{x}^M$, are physical quantities they cannot grow unbound or get negative. For these systems there is thus always a certain perturbation strength that leads to an abrupt break down of robustness.
For many cases this breakdown can be made more explicit, when accounting for conservation equations. For example the active form of a protein y_1 is related to the total concentration by $y^T = y + y_1$, with y the inactive form. Thus, we have $y_1 \leq y^T$. As the rank condition only uses differential forms, the latter inequality has to be additionally fulfilled.

It is further important to note that robustness as defined by the rank condition $rank(\boldsymbol{P}|\boldsymbol{M}|\boldsymbol{K}) = rank(\boldsymbol{M}|\boldsymbol{K})$ does not imply stability of the reaction network. In addition to the formal conditions derived in this report, global stability of a stationary state should also be checked in order to obtain a meaningful notion of robustness. For example, it has to be ensured that the Jacobian matrix has negative eigenvalues for all physical values of reaction fluxes and state variables. Numerically this is most simply tested by showing that the scaled Jacobian matrix $\boldsymbol{N}[diag\,\boldsymbol{v}^s](\boldsymbol{M}|\boldsymbol{A})$ has negative eigenvalues for all positive $\boldsymbol{v}^s$ and all physical values of the unknown entries of $(\boldsymbol{M}|\boldsymbol{A})$.

B. Fine-tuned global robustness

For some reaction networks it might not be clear beforehand if the parameter dependent logarithmic derivatives – denoted by Greek symbols – are indeed independent. In this case there could exist a functional dependence between some of the non-constant elements in the matrix $(\boldsymbol{P}|\boldsymbol{M}|\boldsymbol{K})$ that increases the invariant subspace. An example is given by the following

(rather artificial) system that includes a specific perturbation parameter, $0 < p < 1$,

$$\dot{x} = \underbrace{-k^* \ln(p)}_{v_1} - \underbrace{k^* x}_{v_2} \tag{128}$$

$$\dot{y} = \underbrace{k_1 \exp(-x)}_{v_3} - \underbrace{k_2 y\, p}_{v_4} \tag{129}$$

In the stationary state we can substitute $x^s = -\ln(p)$ into the second equation get $y^s = k_1/k_2$. Thus the systems output is obviously independent of p. Although this systems shows global robustness against p, the reaction network involves fine tuning, in the sense that two independent reactions share the same rate constant, k^*. Using our formalism in a straight forward manner, we arrive, after introducing the independent functions α and β, at

$$rank \left(\begin{array}{c|cccc} & P & X & K_1 & K_2 \\ \hline v_1 & \beta & 0 & 1 & 0 \\ v_2 & 0 & 1 & 1 & 0 \\ v_3 & 0 & \alpha & 0 & 1 \\ v_4 & 1 & 0 & 0 & 1 \end{array} \right) \neq rank \left(\begin{array}{c|ccc} & X & K_1 & K_2 \\ \hline v_1 & 0 & 1 & 0 \\ v_2 & 1 & 1 & 0 \\ v_3 & \alpha & 0 & 1 \\ v_4 & 0 & 0 & 1 \end{array} \right) \tag{130}$$

However, as $\alpha := x^s$, $\beta := [\ln p]^{-1}$ and $x^s = -\ln p$, we see that $\alpha = \beta^{-1}$. After multiplication of the P-column with β^{-1} we see that the rank condition is indeed fulfilled. This fine tuned global robustness can be numerically detected by inserting the actual values for the Greek symbols for one stationary state of the system. If robustness with respect to P differs in this case to randomly chosen Greek symbols, then there exists a hidden dependency among the parameter dependent logarithmic derivatives. This example shows that global robustness of reaction network can also emerge from a combination of fine-tuning and network structure. Here the hyperplane spanned by the column vectors of $(\boldsymbol{M}|\boldsymbol{K})$ is rotating in space and the perturbation, $\boldsymbol{P}$, is such that it constantly follows the rotation of this hyperplane for all $\boldsymbol{p}$. In general, our assumption of independence between the partial derivatives represents a worst-case scenario and is sufficient for global robustness.

C. Numerical test of the rank condition

In practice, a simple numerical test for the rank condition Eq. (6) can be performed by treating the independently varying logarithmic derivatives as random variables. In the case of the example in Section II B this can be realized by redefining the Greek symbols as

random variables that take values according to a uniform distribution $U(-1, 1)$. In doing so, it has to be guaranteed that each randomly chosen value must be sufficiently different from zero and sufficiently different from all other random values. The minimum differences between the random values are set by the numerical precision and avoid that by chance almost equal values are assigned to linear independent logarithmic derivatives.

D. Determination of the parameter-independent nullspace $\boldsymbol{K'}$

So far, the nullspace $\boldsymbol{K}$ – determined by $\boldsymbol{N} \cdot \text{diag}(\boldsymbol{v}^s) \cdot \boldsymbol{K} = \boldsymbol{0}$ – depends on the particular flux distribution $\boldsymbol{v^s}$. To identify the parameter-free conditions for structural robustness, it is desirable to identify the subspace of $\boldsymbol{K}$ that is independent of the particular state $\boldsymbol{v^s}$.
To this end, we first construct the nullspace $\boldsymbol{K}$ from the nullspace of the original stoichiometric matrix, $\boldsymbol{N} \cdot \boldsymbol{K}^N = \boldsymbol{0}$, using the transformation

$$\boldsymbol{K} = [\text{diag}\, \boldsymbol{v}^s]^{-1} \cdot \boldsymbol{K}^N \cdot \boldsymbol{q} \tag{131}$$

where $\boldsymbol{q}$ denotes an invertible $(k - m) \times (k - m)$ matrix, corresponding to a basis transformation of the non-unique representation of the nullspace.
At this point it may be argued that the flux dependency of $\boldsymbol{K}$ can be partially removed by using elementary matrix operations. However, it is far to restrictive to treat the stationary fluxes $\boldsymbol{v}^s = (v_1^s, ..., v_k^s)$ as unknowns as the fluxes are not linear independent

$$\boldsymbol{v}^s = \boldsymbol{K}^N \cdot \boldsymbol{\alpha} \ , \tag{132}$$

with $\boldsymbol{\alpha}$ the elementary flux coefficients and $\dim \boldsymbol{\alpha} = \dim \boldsymbol{v}^s - rank(\boldsymbol{N}) = k - m$. We therefore utilize the freedom of constructing the matrix $\boldsymbol{q}$ in order to generate the largest possible subspace $\boldsymbol{K}^{(1)}$ that is independent of reaction fluxes. We first rewrite Eq. (131) as

$$K_{nm} = \frac{\sum_j K_{nj}^N \, q_{jm}}{\sum_j K_{nj}^N \, \alpha_j}. \tag{133}$$

The parameter independent subspace of $\boldsymbol{K}$ defines $\boldsymbol{K}^{(1)}$ and requires that the elements of $\boldsymbol{K^{(1)}}$ are independent of $\boldsymbol{\alpha}$. This implies the condition

$$\partial_{\alpha_s} K_{nm}^{(1)} = 0 = \partial_{\alpha_s} \frac{\sum_j K_{nj}^N \, q_{jm}}{\sum_j K_{nj}^N \, \alpha_j} = \frac{\sum_j K_{nj}^N \, (\partial_{\alpha_s} q_{jm})}{\sum_j K_{nj}^N \, \alpha_j} - \frac{\sum_j K_{nj}^N \, q_{jm}}{(\sum_j K_{nj}^N \, \alpha_j)^2} K_{is}^N \tag{134}$$

for all indices s, which can be rewritten to give

$$\sum_j K^N_{nj}\,\partial_{\alpha_s} q_{jm} = \underbrace{\frac{\sum_j K^N_{nj}\,q_{jm}}{\sum_j K^N_{nj}\,\alpha_j}}_{const} K^N_{ns} \tag{135}$$

that in turn takes – by defining $\boldsymbol{K}^N_j$ as the j-th column of $\boldsymbol{K}^N$ – the alternative form

$$\sum_j \boldsymbol{K}^N_j\,\partial_{\alpha_s} q_{jm} \propto \boldsymbol{K}^N_s \tag{136}$$

The latter statement requires $q_{jm} \propto \alpha_j$ for all m and thus at least one column vector of $\boldsymbol{q}$ exists, given by $\boldsymbol{q}^* = \boldsymbol{\alpha}$, that results in a parameter free representation of $\boldsymbol{K}$. By Eq. (133) we obtain the first parameter free nullspace vector, $K^{(1)}_{n1} = \sum_j K^N_{nj}\,\alpha_j / \sum_j K^N_{nj}\,\alpha_j = 1$ for all rows, n. A k-dimensional vector consisting only of ones is thus always part of the invariant subspace and reflects an obvious invariance property of all stationary networks: multiplication of all fluxes in the network by the same factor does not change any stationary state variable of the network.

As this invariance property can hold also locally, we can separate $\boldsymbol{q}^*$ in the maximum number of orthogonal column vectors $\boldsymbol{q}^*_1 = (\alpha_1, ..., \alpha_{l_1}, 0, ..., 0)^T$, $\boldsymbol{q}^*_2 = (0, ..., 0, \alpha_{(l_1+1)}, ..., \alpha_{l_2}, 0, ..., 0)^T$, ..., $\boldsymbol{q}^*_z = (0, ..., 0, \alpha_{l_{z-1}+1}, ..., \alpha_{k-m})^T$ such that Eq.(136) holds. The independent columns $\{\boldsymbol{q}^*_1, ..., \boldsymbol{q}^*_z\}$ can be determined by a block matrix representation of $\boldsymbol{K}^N$ which is obtained by resorting columns and rows such that the resulting matrix has only zero entries to all sides of each block. Note that the blocks are in general not square. This resorting leads to

$$\boldsymbol{K}^{(1)} = \begin{bmatrix} \frac{1}{(\boldsymbol{K}^N\cdot\boldsymbol{\alpha})_1} & & 0 \\ & \ddots & \\ 0 & & \frac{1}{(\boldsymbol{K}^N\cdot\boldsymbol{\alpha})_k} \end{bmatrix} \cdot \underbrace{\begin{bmatrix} \boldsymbol{K}^N_1 & & 0 \\ & \ddots & \\ 0 & & \boldsymbol{K}^N_z \end{bmatrix}}_{\boldsymbol{K}^N} \cdot \underbrace{\begin{bmatrix} \boldsymbol{q}^*_1 & & 0 \\ & \ddots & \\ 0 & & \boldsymbol{q}^*_z \end{bmatrix}}_{\boldsymbol{q}^*} \tag{137}$$

$$= \begin{bmatrix} \frac{1}{(\boldsymbol{K}^N\cdot\boldsymbol{\alpha})_1} & & 0 \\ & \ddots & \\ 0 & & \frac{1}{(\boldsymbol{K}^N\cdot\boldsymbol{\alpha})_k} \end{bmatrix} \cdot \begin{bmatrix} \boldsymbol{K}^N_1\cdot\boldsymbol{q}^*_1 & & 0 \\ & \ddots & \\ 0 & & \boldsymbol{K}^N_z\cdot\boldsymbol{q}^*_z \end{bmatrix}. \tag{138}$$

Thus the column vectors of the matrix $\boldsymbol{K}^{(1)}$ that indicate robustness to a fold change in several fluxes can be constructed by the surprisingly simple transformation

$$\mathbf{K}^N = \begin{pmatrix} \boldsymbol{K}_1^N & & 0 \\ & \ddots & \\ 0 & & \boldsymbol{K}_z^N \end{pmatrix} \rightarrow \begin{pmatrix} \mathbf{1} & & 0 \\ & \ddots & \\ 0 & & \mathbf{1} \end{pmatrix} = \mathbf{K}^{(\mathbf{1})} \tag{139}$$

where the $\mathbf{1}$'s denote a vector of ones with dimension set to number of rows of the corresponding block. $\boldsymbol{K}^{(\mathbf{1})}$. Next we complete $\boldsymbol{K}^{(1)}$ and identify the complementary nullspace, $\boldsymbol{K}^{(2)}$, to $\boldsymbol{K}^{(1)}$ such that both spaces together span the complete nullspace of $\boldsymbol{N} \cdot \mathrm{diag}(\boldsymbol{v}^s)$, $\boldsymbol{K} = (\boldsymbol{K}^{(1)}|\boldsymbol{K}^{(2)})$.

We illustrate the construction of $\boldsymbol{K}^{(1)}$ and $\boldsymbol{K}^{(2)}$ by an example of a reaction network which gives rise to two alternative stationary flux distributions. The example consists of two pathways

$$\stackrel{v1}{\rightarrow} A \stackrel{v2}{\rightarrow} B \stackrel{v3}{\rightarrow} C \stackrel{v4}{\rightarrow}$$

and

$$\stackrel{v1}{\rightarrow} A \stackrel{v5}{\rightarrow} D \stackrel{v6}{\rightarrow} C \stackrel{v4}{\rightarrow}$$

The stoichiometric matrix and its corresponding nullspace are given by

$$\boldsymbol{N} = \begin{pmatrix} 1 & -1 & 0 & 0 & -1 & 0 \\ 0 & 1 & -1 & 0 & 0 & 0 \\ 0 & 0 & 1 & -1 & 0 & 1 \\ 0 & 0 & 0 & 0 & 1 & -1 \end{pmatrix} , \quad \boldsymbol{K}^N = \begin{pmatrix} 1 & 1 \\ 1 & 0 \\ 1 & 0 \\ 1 & 1 \\ 0 & 1 \\ 0 & 1 \end{pmatrix} . \tag{140}$$

showing explicitly the two above mentioned flux distributions. The nullspace $\boldsymbol{K}^{(1)}$ consists of one column vector $\boldsymbol{K}^{(1)} = (1\ 1\ 1\ 1\ 1\ 1)^T$ as obviously $\boldsymbol{K}^N$ can be grouped only in one single block. As $\boldsymbol{K} = [\mathrm{diag}(\boldsymbol{v}^s)]^{-1} \cdot \boldsymbol{K}^N$ the i-th row of $\boldsymbol{K}^N$ is weighted by $(v_i^s)^{-1} = (\boldsymbol{K}^N \cdot \boldsymbol{\alpha})_i^{-1}$. As the α_i can vary independently under perturbations, the identity $v_i^s = v_j^s$ holds if the i-th and j-th row of $\boldsymbol{K}^N$ are identical. Identical rows of $\boldsymbol{K}^N$ thus weight the the rows of $\boldsymbol{K}$ the same inverse flux, whereas all other rows of $\boldsymbol{K}$ carry different weights. As the dimension of the joint vector space spanned by $\boldsymbol{K}^{(1)}$ and $\boldsymbol{K}^{(2)}$ must have the same dimension as $\boldsymbol{K}$ – which by construction has the the dimension of $\boldsymbol{K}^N$ – we can choose only one column of $\boldsymbol{K}^N$

to construct $\boldsymbol{K}^{(2)}$ in this example. Taking the second column and indicating the unknown inverse fluxes by Greek symbols we obtain

$$\boldsymbol{K} = \begin{pmatrix} 1 & \beta_1 \\ 1 & 0 \\ 1 & 0 \\ 1 & \beta_1 \\ 1 & \beta_2 \\ 1 & \beta_2 \end{pmatrix} . \tag{141}$$

with $\beta_1 = (v_1^s)^{-1} = (v_4^s)^{-1}$, and $\beta_2 = (v_5^s)^{-1} = (v_6^s)^{-1}$. If we multiply the first column of $\boldsymbol{K}$ by β_2 and subtract the second column we obtain $\beta_2 \boldsymbol{K}_1 - \boldsymbol{K}_2 = (\beta_1' \; \beta_2 \; \beta_2 \; \beta_1' \; 0 \; 0)^T$ with $\beta_1' = \beta_2 - \beta_1$. This result would have been obtained by taking the first column of $\boldsymbol{K}^N$ to construct $\boldsymbol{K}^{(2)}$. We note that $\boldsymbol{K}^{(\mathbf{2})}$ is in general not part of the invariant subspace, due to its dependence on the (local) flux distribution. This dependence is obvious in the example above, where the two column vectors of $\boldsymbol{K}$ span a two dimensional hyperplane and one vector cause the hyperplane to rotate in six dimensional space under a change in stationary flux distributions. The method introduced so far allows to construct in a systematic way the nullspace $\boldsymbol{K}$ such that the dependence on stationary fluxes – that is the number of unknowns β_i – is reduced to a minimum. The subspace spanned by the columns $\boldsymbol{K}$ can be further reduced by elementary matrix operations (EMO)

$$\boldsymbol{K} = \begin{pmatrix} 1 & \beta_1 \\ 1 & 0 \\ 1 & 0 \\ 1 & \beta_1 \\ 1 & \beta_2 \\ 1 & \beta_2 \end{pmatrix} \xrightarrow{EMO} \begin{pmatrix} 1 & 1 \\ 1 & 0 \\ 1 & 0 \\ 1 & 1 \\ 1 & \delta \\ 1 & \delta \end{pmatrix} \tag{142}$$

with $\delta = \beta_2/\beta_1$.

---

[1] Bardwell, L., X. Zou, Q. Nie, and N. L. Komarova (2007, May). Mathematical models of specificity in cell signaling. *Biophys J 92*(10), 3425–3441.

[2] Batchelor, E. and M. Goulian (2003, Jan). Robustness and the cycle of phosphorylation and dephosphorylation in a two-component regulatory system. *Proc Natl Acad Sci U S A 100*(2), 691–696.

[3] Heinrich, R. and S. Schuster (1996). *The regulation of cellular systems*. Chapman & Hall. New York.

[4] Kohanski, M. A. and J. J. Collins (2008, Jun). Rewiring bacteria, two components at a time. *Cell 133*(6), 947–948.

[5] Kollmann, M., L. Løvdok, K. Bartholomé, J. Timmer, and V. Sourjik (2005, Nov). Design principles of a bacterial signalling network. *Nature 438*(7067), 504–507.

[6] Komarova, N. L., X. Zou, Q. Nie, and L. Bardwell (2005). A theoretical framework for specificity in cell signaling. *Mol Syst Biol 1*, 2005.0023.

[7] Ni, X. Y., T. Drengstig, and P. Ruoff (2009, Sep). The control of the controller: molecular mechanisms for robust perfect adaptation and temperature compensation. *Biophys J 97*(5), 1244–1253.

[8] Shinar, G. and M. Feinberg (2010, Mar). Structural sources of robustness in biochemical reaction networks. *Science 327*(5971), 1389–1391.

[9] Shinar, G., R. Milo, M. R. Martínez, and U. Alon (2007, Dec). Input output robustness in simple bacterial signaling systems. *Proc Natl Acad Sci U S A 104*(50), 19931–19935.

[10] Skerker, J. M., B. S. Perchuk, A. Siryaporn, E. A. Lubin, O. Ashenberg, M. Goulian, and M. T. Laub (2008, Jun). Rewiring the specificity of two-component signal transduction systems. *Cell 133*(6), 1043–1054.

[11] Sourjik, V. and H. C. Berg (2000). Localization of components of the chemotaxis machinery of *Escherichia coli* using fluorescent protein fusions. *Mol. Microbiol. 37*, 740–751.

[12] Sourjik, V. and H. C. Berg (2002, Jan). Receptor sensitivity in bacterial chemotaxis. *Proc Natl Acad Sci U S A 99*(1), 123–127.

[13] Sourjik, V. and H. C. Berg (2004). Functional interactions between receptors in bacterial chemotaxis. *Nature 428*, 437.

[14] Sourjik, V., A. Vaknin, T. S. Shimizu, and H. C. Berg (2007). In vivo measurement by FRET of pathway activity in bacterial chemotaxis. *Methods Enzymol 423*, 365–391.

[15] Vecchio, D. D., A. J. Ninfa, and E. D. Sontag (2008). Modular cell biology: retroactivity and insulation. *Mol Syst Biol 4*, 161.
